# Immunoprofiling reveals cell subsets associated with the trajectory of cytomegalovirus reactivation post stem cell transplantation

**DOI:** 10.1038/s41467-022-29943-9

**Published:** 2022-05-11

**Authors:** Lauren Stern, Helen M. McGuire, Selmir Avdic, Barbara Fazekas de St Groth, David Gottlieb, Allison Abendroth, Emily Blyth, Barry Slobedman

**Affiliations:** 1 School of Medical Sciences, Faculty of Medicine and Health, The University of Sydney, Sydney, Australia; 2 Charles Perkins Centre, The University of Sydney, Sydney, Australia; 3 Ramaciotti Facility for Human Systems Biology, The University of Sydney, Sydney, Australia; 4 Westmead Institute for Medical Research, Faculty of Medicine and Health, The University of Sydney, Sydney, Australia; 5 Blood and Marrow Transplant Unit, Westmead Hospital, Sydney, Australia

**Keywords:** Viral infection, Herpes virus, Transplant immunology, Infection

## Abstract

Human cytomegalovirus reactivation is a major opportunistic infection after allogeneic haematopoietic stem cell transplantation and has a complex relationship with post-transplant immune reconstitution. Here, we use mass cytometry to define patterns of innate and adaptive immune cell reconstitution at key phases of human cytomegalovirus reactivation in the first 100 days post haematopoietic stem cell transplantation. Human cytomegalovirus reactivation is associated with the development of activated, memory T-cell profiles, with faster effector-memory CD4^+^ T-cell recovery in patients with low-level versus high-level human cytomegalovirus DNAemia. Mucosal-associated invariant T cell levels at the initial detection of human cytomegalovirus DNAemia are significantly lower in patients who subsequently develop high-level versus low-level human cytomegalovirus reactivation. Our data describe distinct immune signatures that emerged with human cytomegalovirus reactivation after haematopoietic stem cell transplantation, and highlight Mucosal-associated invariant T cell levels at the first detection of reactivation as a marker that may be useful to anticipate the magnitude of human cytomegalovirus DNAemia.

## Abstract (web summary)

Human cytomegalovirus is a major cause of morbidity and mortality in transplant patients and multiple immune cells types are critical during infection and reactivation. Here the authors assess the immune cell compartments of haematopoietic stem cell recipients in the early period post transplantation and identify key features of effector memory CD4^+^ T cells and mucosal associated invariant T cells in this context.

## Introduction

Human cytomegalovirus (HCMV) reactivation is a major complication after allogeneic haematopoietic stem cell transplantation (HSCT)^1,2^. It is associated with significant morbidity, increased non-relapse mortality, microbial superinfections and acute graft-versus-host disease (aGvHD)^3–6^. Higher HCMV viraemia levels and area under the curve (AUC) of viral load are linked to increased risk of mortality post-HSCT^5,7^. A dose-response relationship between the magnitude of HCMV viraemia and risk of mortality has been reported^5^. While antiviral prophylaxis or pre-emptive therapy (initiated following detection of HCMV DNAemia) can reduce the incidence of end-organ HCMV disease, commonly used antiviral drugs such as ganciclovir and foscarnet carry significant toxicities^8^. Improved risk stratification methods are needed to enable early intervention in patients who will develop high-level HCMV viraemia, while minimising unnecessary exposure to antivirals in those who experience low-level, self-limiting HCMV reactivation.

Immune reconstitution after HSCT has a close relationship with HCMV reactivation^9^. Measurements of immune reconstitution, such as HCMV-specific T cell responses, may be integrated alongside viral load monitoring to guide the targeting of antiviral therapy^10–18^. High-dimensional technologies such as mass cytometry (CyTOF)^19,20^, which utilises antibodies conjugated to heavy-metal isotopes to allow simultaneous assessment of > 40 single-cell markers, have the potential to aid in identification of clinically important immune signatures and improve our understanding of the complex relationship between HCMV reactivation, immune reconstitution and clinical outcome^21^.

Herein, we used CyTOF to investigate the dynamics of innate and adaptive immune cell reconstitution in the first 100 days post-transplant in a cohort of 35 adult allogeneic HSCT patients who experienced high-level, low-level or no HCMV reactivation. Comprehensive immunophenotyping was performed on peripheral blood samples from four time-points aligning with the kinetics of HCMV DNAemia (prior to detection, initial detection, peak, and near the resolution). Our results identified mucosal-associated invariant T (MAIT) cells and effector-memory (EM) CD4^+^ T cells as key features distinguishing patients with low-level and high-level HCMV reactivation, and provide a detailed analysis of patterns of immune recovery accompanying different phases of HCMV reactivation in the first 100 days post-HSCT.

## Results

### Patient characteristics

**Fig. 1:**
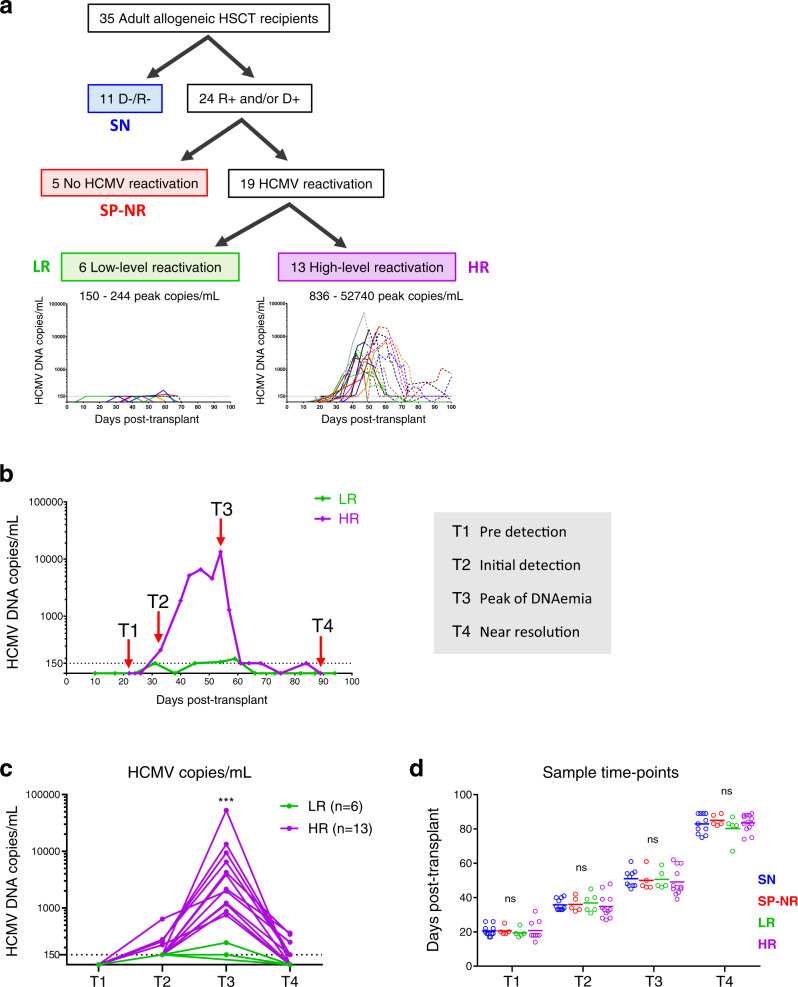
Study design. **a** HSCT recipients were divided into four groups: SN (HCMV-seronegative D-/R-; blue), SP-NR (HCMV-seropositive R + and/or D + , with no detected HCMV DNAemia in the first 100 days post-HSCT; red), LR (patients who developed low-level (<250 peak copies/mL) HCMV DNAemia; green), and HR (patients who developed high-level (> 830 peak copies/mL) HCMV DNAemia; purple). Graphs show HCMV DNA copies/mL plasma in the first 100 days post-transplant for LR (left) and HR (right). Each line represents one patient. The point of antiviral therapy initiation for each patient is indicated by the shift from solid line (pre-therapy) to dotted line (after therapy initiation). The horizontal dotted line at 150 copies/mL indicates the lower limit of quantitation (LLQ). **b** Representative HCMV reactivation profiles from one HR and one LR patient depicting the four time-points analysed with mass cytometry. Peripheral blood mononuclear cells (PBMC) collected prior to detection (T1), at the initial detection (T2), at the peak (T3) and near the resolution (T4) of HCMV DNAemia post-HSCT were analysed. The horizontal dotted line indicates the LLQ (150 HCMV DNA copies/mL). Samples from corresponding days post-transplant were analysed from HSCT recipients without HCMV reactivation. **c** HCMV DNA plasma copy load at each study time-point from patients with HCMV reactivation. Each line represents a patient. The horizontal dotted line at 150 copies/mL indicates the LLQ. Two-tailed Mann-Whitney test comparing HR and LR per time point (***p = 0.0003). **d** Days post-transplant for all PBMC samples analysed with mass cytometry. Lines indicate the mean. One-way ANOVA per time-point with Fisher’s Least Significant Difference test (ns, not significant). T1 (SN *n* = 11, SP-NR *n* = 5, LR *n* = 5, HR *n* = 9), T2 (SN *n* = 11, SP-NR *n* = 5, LR *n* = 6, HR *n* = 12), T3 (SN *n* = 10, SP-NR *n* = 5, LR *n* = 5, HR *n* = 12), T4 (SN *n* = 11, SP-NR *n* = 5, LR *n* = 5, HR *n* = 13). SN HCMV-seronegative, SP-NR Seropositive no reactivation, LR Low-level HCMV reactivation, HR High-level HCMV reactivation, D Donor, R Recipient, HCMV human cytomegalovirus, HSCT haematopoietic stem cell transplant. Source data are provided as a Source Data file.

We studied 35 allogeneic HSCT recipients in the first 100 days post-transplant (Fig. 1a). Patient characteristics are outlined in Table 1. Of the 24 patients at risk of HCMV reactivation (i.e., HCMV-seropositive donor (D + ) and/or recipient (R + )), HCMV reactivation was detected in 19 (79.2%) patients, at a median 29 (12–46) days post-HSCT. Two patterns of HCMV reactivation were observed (Fig. 1a); low-level reactivation (LR; < 250 peak HCMV DNA copies/mL; *n* = 6) and high-level reactivation (HR; > 830 peak copies/mL; *n* = 13). We thus retrospectively divided the cohort into four study groups (Fig. 1a): LR (low-level HCMV reactivation; *n* = 6), HR (high-level HCMV reactivation; *n* = 13), SP-NR (HCMV-seropositive D + and/or R + , with no documented HCMV reactivation; *n* = 5), and SN (D-/R- HCMV-seronegative, with no HCMV infection; *n* = 11). Peak HCMV DNAemia levels were significantly higher in HR (median 3873 (836-52740) copies/mL) compared to LR (150 (150-244) copies/mL; *p* < 0.0001) patients (Supplementary Fig. 1A). No patient was diagnosed with HCMV end-organ disease in the follow-up period.

**Table 1:** Characteristics of study subjects.

Characteristic	All patients (*n* = 35)	SN (*n* = 11)	SP-NR (*n* = 5)	LR (*n* = 6)	HR (*n* = 13)	LR v HR *p* value	SP-NR v R (LR, HR) *p* value	R v NR^a^ *p* value
Demographics								
Age (median (range)), years	47 (18–70)	44 (23–70)	59 (19–60)	42.5 (24–62)	59 (18–67)	0.3554	0.9312	0.6291
Sex (M:F)	20: 15	10: 1	3: 2	2: 4	5: 8	>0.9999	0.6146	**0.0155**
*HCMV serostatus (n)*								
D-/R-	11	11	0	0	0	0.1280	ND	ND
D + /R-	3	0	3	0	0			
D-/R +	5	0	0	0	5			
D + /R +	16	0	2	6	8			
*Underlying disease (n)*								
AML	19	9	2	4	4	ND	ND	ND
ALL	7	0	2	1	4			
MDS	3	0	1	0	2			
SAA	3	0	0	1	2			
MPD	2	1	0	0	1			
Lymphoma	1	1	0	0	0			

Transplant characteristics								
*Conditioning (n)*								
MAC	14 (40%)	6 (55%)	2 (40%)	3 (50%)	3 (23%)	0.3201	>0.9999	0.3167
RIC	21 (60%)	5 (45%)	3 (60%)	3 (50%)	10 (77%)			
*Stem cell source (n)*								
Peripheral blood	32 (91%)	11 (100%)	5 (100%)	5 (83%)	11 (85%)	>0.9999	>0.9999	0.2336
Bone marrow	3 (9%)	0	0	1 (17%)	2 (15%)			
*Donor type (n)*								
MUD	22	7	3	3	9	ND	ND	ND
Haploidentical	4	1	1	0	2			
HLA-identical related	9	3	1	3	2			
T cell depletion *(n)*	20 (57%)	4 (36%)	2 (40%)	4 (67%)	10 (77%)	>0.9999	0.2885	**0.0442**
ATG	17	4	2	3	8			
Alemtuzumab	3	0	0	1	2			

Post transplant events								
HCMV reactivation, *n* (%)	19 (54%)	0	0	6 (100%)	13 (100%)	ND	ND	ND
EBV reactivation, *n* (%)	17 (49%)	6 (55%)	1 (20%)	1 (17%)	9 (69%)	0.0573	0.3271	0.7380
*Acute GvHD*, *n* (%)								
Overall (grade II–IV)	9 (26%)	4^b^ (36%)	1 (20%)	0	4 (31%)	0.2554	>0.9999	0.7003
Severe (grade III–IV)	2 (6%)	2 (18%)	0	0	0			
Relapse, *n* (%)	11 (31%)	3 (27%)	2 (40%)	1 (17%)	5 (38%)	0.6047	>0.9999	>0.9999
Death (1^st^ year post-HSCT), *n* (%)	4 (11%)	0	1 (20%)	0	3 (23%)	0.5170	>0.9999	0.6081
Death (overall follow up), *n* (%)	7 (20%)	0	1 (20%)	1 (17%)	5 (38%)	0.6047	>0.9999	0.0964
Length of follow up (median (range)), days	786 (77–1323)	931 (448–1323)	777 (149–1011)	764 (77–1043)	592 (78–1242)	0.9661	0.7306	**0.0263**

^a^R (HCMV reactivators, i.e., LR + HR; *n* = 19), NR (non-reactivators, i.e., SN + SPNR; *n* = 16). ^b^ Includes *n* = 1 grade unknown.

Age and sex refer to the HSCT recipient. Data are presented as absolute values or median values with range. Two-tailed Mann-Whitney test for continuous variables, two-sided Fisher’s exact test for categorical variables (p < 0.05 in bold). ALL (acute lymphoblastic leukaemia), AML (acute myeloid leukaemia), ATG (anti-thymocyte globulin), D (donor), EBV (Epstein-Barr Virus), GvHD (graft-versus-host disease), HCMV (human cytomegalovirus), HR (high-level HCMV reactivation; 830-53,000 peak HCMV DNA copies/mL), HSCT (haematopoietic stem cell transplant), LR (low-level HCMV reactivation; <250 peak HCMV DNA copies/mL), MAC (myeloablative conditioning), MDS (myelodysplastic syndrome), MPD (myeloproliferative disorder), MUD (matched unrelated donor), ND (not determined), R (recipient), RIC (reduced intensity conditioning), SAA (severe aplastic anaemia), SN (HCMV seronegative; D-/R-), SP-NR (HCMV seropositive no reactivation).

Bold values indicate statistical significance *p* < 0.05.

Previous studies have noted that HCMV reactivation after HSCT is associated with complications such as aGvHD, Epstein-Barr Virus (EBV) reactivation and poorer survival^3,5,22^. Overall survival in our cohort was 88.6% (31/35) in first year post-transplant (Table 1). Both patients who died in the first 100 days, and 3/4 patients who died in the first year post-transplant, were HR patients (additional patient was SP-NR). There was a higher incidence of EBV reactivation in HR vs. LR groups (9/13 (69.2%) vs. 1/6 (16.7%); *p* = 0.0573), and the incidence of aGvHD grades II-IV was also higher in HR vs. LR (4/13 vs. 0/6; *p* = 0.2554), although this was not statistically significant (Table 1).

### Features of HCMV reactivation

The median day and magnitude of first detected HCMV DNAemia did not differ significantly between LR and HR (Supplementary Table 1). However, as expected, HR had a longer duration of HCMV DNAemia than LR (median 60 (28–121) vs. 21.5 (7–49) days, respectively; *p* = 0.0180), and the log_10_ area under the curve of HCMV DNA copies/mL (a measure of HCMV antigen exposure) in the first 100 days post-HSCT (AUC_0-100_) was significantly higher in HR than LR (Supplementary Fig. 1b). All (13/13) HR patients received pre-emptive antiviral therapy to control HCMV reactivation, whereas a majority of LR patients (5/6) had self-limiting HCMV DNAemia (*p* = 0.0005). The one LR patient who received pre-emptive antiviral therapy had low HCMV DNAemia levels consistent with the other LR patients (Fig. 1a). Resolution of HCMV reactivation by day 100 post-HSCT was achieved in 6/6 (100%) LR patients, compared to 6/13 (46.2%) HR patients (*p* = 0.0436).

### Increased CD8^+^ T cell number and inverted CD4:CD8 ratio after HCMV reactivation

To comprehensively describe immune reconstitution at key time-points during HCMV reactivation post-HSCT, we employed CyTOF to analyse PBMC samples collected prior to detection (T1), at initial detection (T2), at peak (T3) and near resolution (T4) of HCMV DNAemia in the first 100 days post-HSCT (Fig. 1b; Supplementary Table 2). We designed a panel of 36 heavy metal isotope-tagged antibodies (Supplementary Table 3) targeted at innate and adaptive PBMC populations, allowing identification of 77 subsets at each time-point (see Supplementary Table 4; Supplementary Table 5; Supplementary Fig. 2). No HCMV-specific peptide-MHC tetramers were included. HCMV DNA copy numbers per time-point are shown in Fig. 1c. Samples from matched days post-transplant were analysed in parallel from HSCT recipients who did not experience HCMV reactivation (SN and SP-NR) (Fig. 1d).

**Fig. 2:**
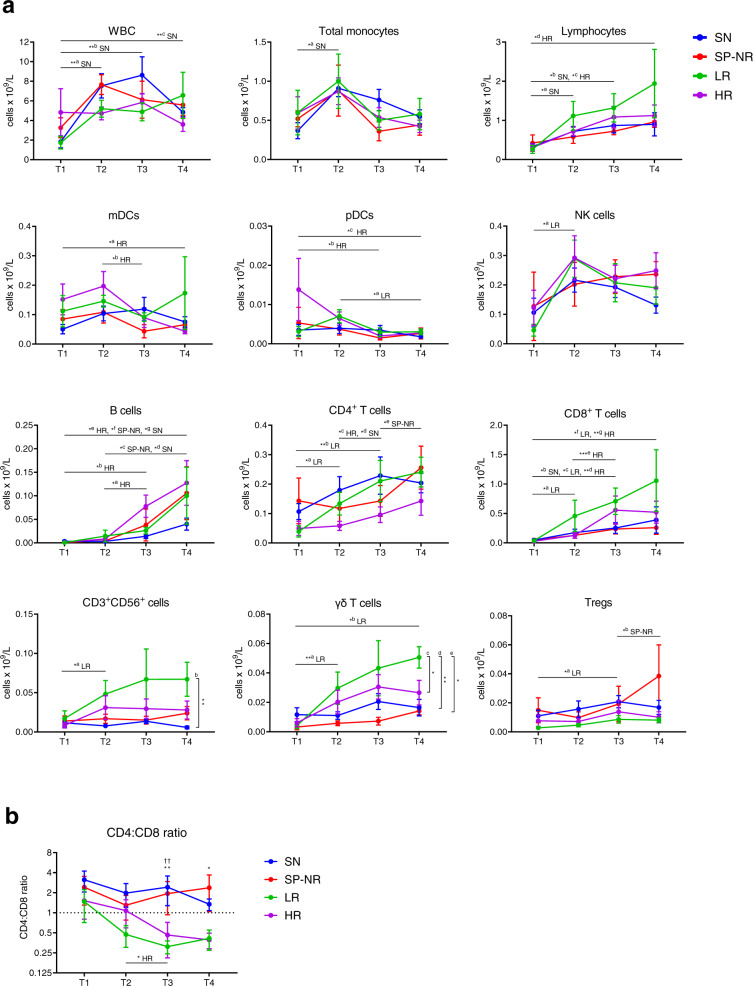
Reconstitution kinetics of peripheral blood mononuclear cell populations in the first 100 days post-HSCT. **a** Absolute blood counts (x10^9^/L) of major immune cell subsets over the four study time-points in HSCT recipients with (LR, HR) or without (SN, SP-NR) HCMV reactivation. T1 is prior to detection of HCMV reactivation; T2, at initial detection of HCMV reactivation; T3, the peak; T4, near the resolution of HCMV reactivation. Blood samples from corresponding days post-HSCT were assessed in parallel from HSCT recipients without HCMV reactivation. Mean ± SEM is shown. Statistically significant comparisons are indicated (two-way mixed-effects model with Tukey’s multiple comparisons test applied to log-transformed data; *p < 0.05, **p < 0.01, ***p < 0.001). Horizontal lines indicate significant differences between pairs of time-points for the patient group(s) listed. Vertical brackets indicate significant differences between patient groups at T4. WBC: ^a^*p* = 0.0020, ^b^*p* = 0.0045, ^c^*p* = 0.0041. Monocytes: ^a^*p* = 0.0286. Lymphocytes: ^a^*p* = 0.0440, ^b^*p* = 0.0352, ^c^*p* = 0.0431, ^d^*p* = 0.0222. mDC: ^a^*p* = 0.0477, ^b^*p* = 0.0139. pDC: ^a^*p* = 0.0113, ^b^*p* = 0.0186, ^c^*p* = 0.0459. NK cells: ^a^*p* = 0.0347. B cells: ^a^*p* = 0.0177, ^b^*p* = 0.0366, ^c^*p* = 0.0430, ^d^*p* = 0.0137, ^e^*p* = 0.0159, ^f^*p* = 0.0296, ^g^*p* = 0.0326. CD4^+^ T cells: ^a^*p* = 0.0250, ^b^*p* = 0.0099, ^c^*p* = 0.0329, ^d^*p* = 0.0393, ^e^*p* = 0.0326. CD8^+^ T cells: ^a^*p* = 0.0377, ^b^*p* = 0.0166, ^c^*p* = 0.0328, ^d^*p* = 0.0042, ^e^*p* = 0.0003, ^f^*p* = 0.0205, ^g^*p* = 0.0018. CD3^+^CD56^+^ cells: ^a^*p* = 0.0213, ^b^*p* = 0.0028. γδ T cells: ^a^*p* = 0.0077, ^b^*p* = 0.0336, ^c^*p* = 0.0386, ^d^*p* = 0.0019, ^e^*p* = 0.0142. Tregs: ^a^*p* = 0.0498, ^b^*p* = 0.0347. **b** CD4:CD8 T cell ratio. Mean ± SEM is shown. Two-way mixed-effects model with Tukey’s multiple comparisons test after log-transformation. Horizontal line indicates significant difference between T2 and T3 in HR (*p* = 0.0250). T3: ^††^*p* = 0.0100 for SN vs. LR; **p = 0.0045 for HR vs. SN. T4: *p = 0.0104 for HR vs. SN. For T1, T2, T3 and T4 respectively, SN (*n* = 11, *n* = 11, *n* = 10, *n* = 11), SP-NR (*n* = 5 for all), LR (*n* = 5, *n* = 6, *n* = 5, *n* = 5), HR (*n* = 9, *n* = 12, *n* = 12, *n* = 13). SN HCMV-seronegative, SP-NR Seropositive no reactivation, LR Low-level HCMV reactivation, HR high-level HCMV reactivation. mDC Myeloid dendritic cell, NK Natural killer, pDC plasmacytoid dendritic cell, Treg T regulatory cell, WBC White blood cells. Source data are provided as a Source Data file.

There were no significant differences in the absolute counts of major PBMC subsets between the groups at T1 (Fig. 2a). Subsequently, faster numerical recovery of CD8^+^ T cells, CD3^+^CD56^+^ cells and γδ T cells was observed in patients with HCMV reactivation. Recovery dynamics of innate populations including monocytes and NK cells were similar in all patient groups (Fig. 2a). LR patients demonstrated the fastest reconstitution of CD8^+^ T cells, CD3^+^CD56^+^ cells and γδ T cells, culminating in significantly higher γδ T cell and CD3^+^CD56^+^ cell counts at T4. Significant increases in NK cells, CD3^+^CD56^+^ cells, γδ T cells, CD8^+^ T cells and CD4^+^ T cells between T1 and T2 were evident only in LR patients. HR patients displayed the slowest CD4^+^ T cell recovery, but showed clear increases in B cells and CD8^+^ T cells at T3. Inversion of the CD4:CD8 T cell ratio is a feature previously described in patients who experience HCMV reactivation post-HSCT^23^. As shown in Fig. 2b, we observed that the CD4:CD8 T cell ratio became inverted in HCMV reactivators, diverging at T2 in LR and T3 in HR, while patients without HCMV reactivation maintained a CD4 bias across the time-points (Fig. 2b).

### Distinct pattern of immune reconstitution in patients with HCMV reactivation

**Fig. 3:**
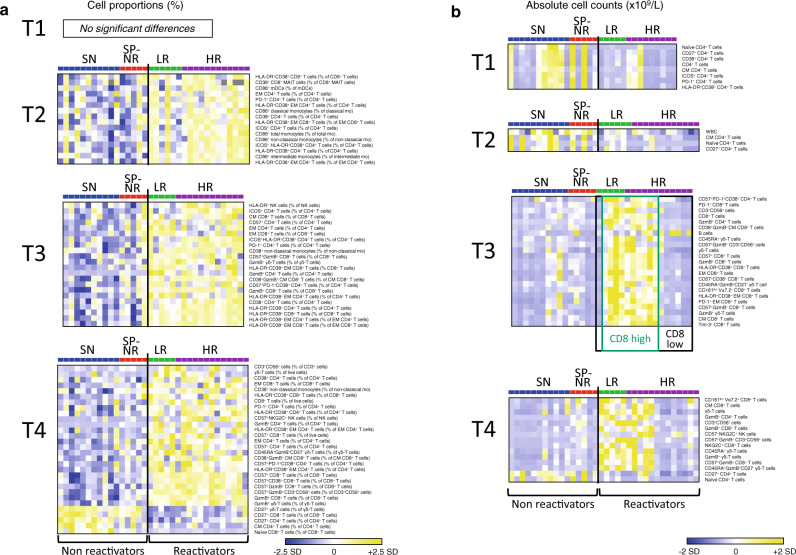
Distinct immune reconstitution pattern in HSCT recipients with HCMV reactivation. **a** Heat map rows display percentages of immune cell subsets that were significantly different between HSCT recipients who did (LR, HR) or did not (SN, SP-NR) experience HCMV reactivation in the first 100 days post-transplant. Percentages are expressed as percent of live cells and/or percent of parent subset. Significance was determined using two-class unpaired significance analysis of microarrays (SAM) involving 88 cell subsets per time-point (see Supplementary Table 4). Heat maps are coloured by the Z-score normalised per row (cell subset). Grey colour indicates missing data (too few cells in parent gate to accurately gate subpopulation). Each column represents a patient. **b** Heat map rows display cell subsets (absolute cell counts; x10^9^/L blood) that differ significantly between HCMV reactivators (LR, HR) and non-reactivators (SN, SP-NR) as determined by two-class unpaired SAM analysis involving 77 populations per time-point (see Supplementary Table 5). For T3 in (**b**), a FDR of 3.614% was used (0.831/23 subsets shown are false discoveries). Heat maps are coloured by the Z-score normalised per row (cell subset). Each column represents a patient. Patients with HCMV reactivation were subsequently stratified into ‘CD8^high^’ (dark green box) or ‘CD8^low^’ (black box) groups according to the presence or absence of the elevated CD8^+^ T cell dominated profile observed at T3. See Supplementary Fig. 3 for a schematic representation of cell subsets shown in Fig. 3a, b. T1 is prior to the detection of HCMV reactivation; T2, at the initial detection of HCMV reactivation; T3, the peak; T4, near the resolution of HCMV reactivation. Naive (CD45RA^+^ CD45RO^-^ CCR7^+^ CD27^+^), CM (central memory; CD45RA^-^ CD45RO^+^ CCR7^+^ CD27^+^), EM (effector memory; CD45RA^-^ CD45RO^+^ CCR7^-^ CD27^-^), Mo (monocyte). T1 (SN *n* = 11, SP-NR *n* = 5, LR *n* = 5, HR *n* = 9), T2 (SN *n* = 11, SP-NR *n* = 5, LR *n* = 6, HR *n* = 12), T3 (SN *n* = 10, SP-NR *n* = 5, LR *n* = 5, HR *n* = 12), T4 (SN *n* = 11, SP-NR *n* = 5, LR *n* = 5, HR *n* = 13). SN HCMV-seronegative (blue), SP-NR Seropositive no reactivation (red), LR Low-level HCMV reactivation (green), HR High-level HCMV reactivation (purple), SD Standard deviation. Source data are provided as a Source Data file.

To detail the immune profile at each time-point, we used significance analysis of microarrays (SAM) to compare all immune subsets analysed between patients with or without HCMV reactivation, examining both cell proportions (Fig. 3a) and absolute counts (Fig. 3b). At T1, absolute counts of total CD4^+^ T cells and CD4^+^ T cell subsets appeared lower in ‘reactivators’ (LR and HR) compared to ‘non-reactivators’ (SN and SP-NR) (Fig. 3b), while no significant differences in cell proportions were identified between these groups (Fig. 3a). At T2, however, multiple cell phenotypes indicative of immune activation were enriched in reactivators at T2 (Fig. 3a), including elevated percentages of CD86^+^ monocyte subsets and myeloid dendritic cells (mDC), HLA-DR^+^CD38^+^ total and EM CD4^+^ and CD8^+^ T cells, and higher EM, PD-1^+^, and ICOS^+^ percentages in the CD4^+^ T cell compartment (Fig. 3a). Total white blood cell (WBC) counts and absolute counts of central-memory (CM), CD27^+^ and naïve CD4^+^ T cells were also observed to be lower in reactivators compared to non-reactivators at T2 (Fig. 3b).

At the peak of HCMV DNAemia (T3), significantly higher percentages of CD4^+^, CD8^+^ and γδ T cells expressing granzyme-B (GzmB) and/or CD57 emerged in reactivators relative to non-reactivators, and remained elevated at T4 (Fig. 3a). These markers (GzmB, CD57) indicative of cytotoxic potential have previously been noted to be expressed by HCMV-specific T cells^23–27^. Higher proportions of CM CD8^+^ T cells, EM CD8^+^ T cells and EM CD4^+^ T cells were also detected in reactivators compared to non-reactivators at T3 (Fig. 3a).

At T4, patients who experienced HCMV reactivation retained a distinct immune profile relative to non-reactivators, dominated by elevated frequencies of activated (HLA-DR^+^CD38^+^), CD57^+^ and GzmB^+^ T cell subsets (Fig. 3a). A majority (16/22; 73%) of the cell subsets detected at significantly higher percentages in reactivators at T3 remained so at T4 (Fig. 3a; Supplementary Fig. 3a). At T4, significantly lower percentages of CD27-expressing T cells, including naïve CD8^+^ T cells and CM CD4^+^ T cells, were detected in reactivators compared to non-reactivators (Fig. 3a), as well as higher proportions of CD57^+^NKG2C^+^ NK cells, γδ T cells and CD3^+^CD56^+^ cells in reactivators (Fig. 3a).

Overall, these results indicate an early activation of the immune environment in patients who develop HCMV reactivation, with elevated proportions of activated myeloid and CD4^+^ T cell subsets at the initial detection of HCMV DNAemia, followed by an accumulation of activated and cytotoxic T cell phenotypes at the peak of reactivation, and sustained memory skewing in the T cell compartment, in comparison to patients who did not experience HCMV reactivation.

### Elevated CD8^+^ T cell dominated signature at the peak of HCMV reactivation in a subset of reactivators

Comparison of absolute cell counts between reactivators and non-reactivators exposed two patterns of quantitative immune recovery within the reactivator group at T3 and T4 (Fig. 3b). A CD8^+^ T cell dominated signature (‘CD8^high^’) comprising significantly higher absolute numbers of activated and memory CD8^+^ T cells, as well as γδ T cell, CD3^+^CD56^+^ cell and CD4^+^ T cell subsets, was evident at T3 in most (4/5) of LR patients and half (6/12) of HR patients (Fig. 3b). At T4, this elevated quantitative immune profile contained 11/23 populations seen at T3, as well as CD57^+^NKG2C^+^ NK cells and NKG2C^+^CD8^+^ T cells (Fig. 3b; Supplementary Fig. 3b, c). Absolute numbers of CD27^+^ CD4^+^ T cells and naïve CD4^+^ T cells were also seen to be significantly lower in HCMV reactivators compared to non-reactivators at T4 (Fig. 3b).

**Fig. 4:**
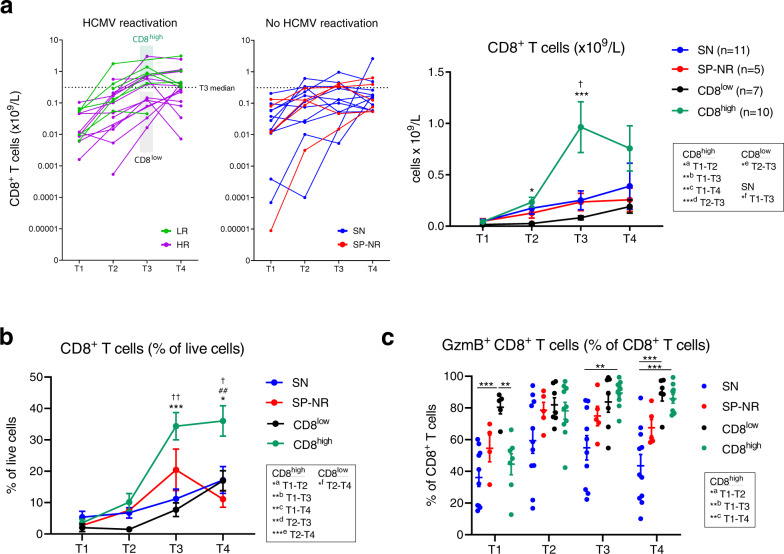
Emergence of a CD8^+^ T cell dominated immune profile in a subset of HSCT recipients with HCMV reactivation. **a** Absolute CD8^+^ T cell counts (x10^9^/L) in HSCT recipients with or without HCMV reactivation. Each line represents one patient. Dotted line indicates median CD8^+^ T cells across all patients at T3 (0.3116 × 10^9^/L). Reactivators were partitioned into ‘CD8^high^’ (*n* = 10) and ‘CD8^low^’ (*n* = 7) groups according to a CD8^+^ T cell-dominated signature at T3 in Fig. 3b. Graph on right: mean ± SEM, two-way mixed-effects model with Tukey’s multiple comparisons tests after log transform. On graph: *p = 0.0153 and ***p = 0.0002 for CD8^high^ (dark green) vs. CD8^low^ (black); ^†^*p* = 0.0143 CD8^high^ vs. SN (HCMV-seronegative). Significant differences between pairs of time-points for patient groups are boxed: ^a^*p* = 0.0118, ^b^*p* = 0.0017, ^c^*p* = 0.0043, ^d^*p* = 0.0009, ^e^*p* = 0.0384, ^f^*p* = 0.0166. For T1, T2, T3 and T4 respectively: CD8^high^ (*n* = 7, *n* = 10, *n* = 10, *n* = 10), CD8^low^ (*n* = 5, *n* = 7, *n* = 7, *n* = 6), SN (*n* = 11, *n* = 11, *n* = 10, *n* = 11), SP-NR (*n* = 5 for all), in (**a**; right graph) and (**b**). **b** CD8^+^ T cells (percentage of live cells) in CD8^high^ reactivators (dark green), CD8^low^ reactivators (black), SN (blue) and SP-NR (seropositive no reactivation; red). Mean ± SEM. On graph: CD8^high^ vs. CD8^low^ (***p = 0.0005, *p = 0.0249), CD8^high^ vs. SN (^††^*p* = 0.0025, ^†^*p* = 0.0414), CD8^high^ vs. SP-NR (^##^*p* = 0.0029). In box adjacent: ^a^*p* = 0.0400, ^b^*p* = 0.0071, ^c^*p* = 0.0016, ^d^*p* = 0.0022, ^e^*p* = 0.0009, ^f^*p* = 0.0177. In (**b**, **c**), two-way mixed-effects models with Tukey’s multiple comparisons tests. **c** Percentage of Granzyme B (GzmB^+^) CD8^+^ T cells in CD8^high^ reactivators, CD8^low^ reactivators, SN and SP-NR. Mean ± SEM shown. On graph: at T1 (**p = 0.0052, ***p = 0.0002), at T3 (**p = 0.0063), at T4 (CD8^low^ vs. SN, ***p = 0.0004; CD8^high^ vs. SN, ***p = 0.0005). In box: ^a^*p* = 0.0446, ^b^*p* = 0.0021, ^c^*p* = 0.0027. For T1, T2, T3 and T4 respectively: CD8^high^ (*n* = 7, *n* = 10, *n* = 10, *n* = 10), CD8^low^ (*n* = 5, *n* = 7, *n* = 7, *n* = 6), SN (*n* = 10, *n* = 11, *n* = 10, *n* = 11), SP-NR (*n* = 4, *n* = 5, *n* = 5, *n* = 5). Reactivators without a T3 sample (*n* = 1 LR, *n* = 1 HR) are not shown in (**a**; right graph), (**b**) or (**c**). LR Low-level reactivation, HR High-level reactivation, T1 Before detection, T2 Initial detection, T3 Peak, and T4 Near resolution, of reactivation. Source data are provided as a Source Data file.

Direct comparison of all cell-subset counts between reactivators with the ‘CD8^high^’ or ‘CD8^low^’ signature at the peak of HCMV reactivation revealed clear differences in numeric T cell recovery between CD8^high^ and CD8^low^ reactivators at T2, T3 and T4 (Supplementary Fig. 4A). In particular, CD8^high^ patients displayed faster recovery of total CD8^+^ T cell absolute counts and percentages over the course of HCMV reactivation (Fig. 4a, b), while CD8^+^ T cell frequencies between CD8^high^ and CD8^low^ patients did not differ significantly before the detection of HCMV reactivation (T1) (Fig. 4a, b). At the peak of HCMV DNAemia (T3), total CD8^+^ T cell counts were significantly higher in CD8^high^ reactivators compared to CD8^low^ reactivators (median 0.7634 vs. 0.07837 × 10^9^/L, respectively; *p* = 0.0004) and non-reactivators (median 0.1291 × 10^9^/L; *p* = 0.0027), but there was no significant difference in CD8^+^ T cell count between non-reactivators and CD8^low^ reactivators (Fig. 4a; Supplementary Fig. 4B). These findings highlight differences in the response to HCMV reactivation and provide evidence indicating that enhanced CD8^+^ T cell recovery is not a universal feature in HSCT recipients at the peak of HCMV reactivation.

The CD8^high^ signature was more common in LR than HR, however there was no significant relationship with viral magnitude or days post-reactivation (Supplementary Table 6). All CD8^low^ reactivators had received T cell depletion (TCD), whereas 6/10 CD8^high^ reactivators underwent TCD. The CD8^low^ group uniquely contained three patients who received bone-marrow grafts and one who experienced rejection (Supplementary Table 6). These factors are associated with slower T cell recovery^28–31^, thus the CD8^low^ signature may be reflective of conditioning and/or stem cell source.

We found that CD8^low^ patients had higher proportions of GzmB^+^ CD8^+^ T cells before the detection of HCMV reactivation (T1), compared to all other patient groups (Fig. 4c). Thus, early enrichment of GzmB^+^ CD8^+^ T cells (above ~74% of CD8^+^ T cells) may be a feature that heralds later development of HCMV reactivation and slower numeric CD8^+^ T cell recovery, particularly in TCD patients.

### MAIT cell frequencies at the initial detection of HCMV reactivation distinguish low-level and high-level reactivators

To further examine immune reconstitution differences between LR and HR patients, we performed SAM comparing the frequencies of all immune subsets examined between LR and HR. This revealed recovery of MAIT cells and EM CD4^+^ T cells as key features distinguishing LR from HR patients (Supplementary Fig. 5).

**Fig. 5:**
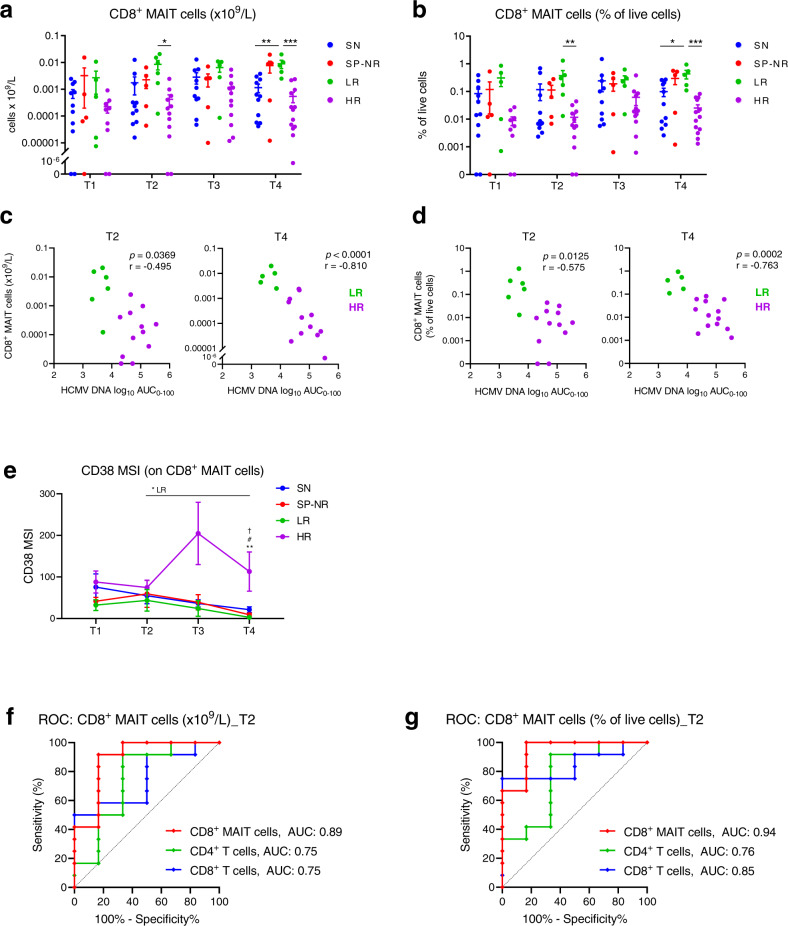
CD8^+^ MAIT cell frequencies at the initial detection of reactivation distinguish low-level and high-level HCMV reactivators. CD8^+^ MAIT cells were identified as Vα7.2^+^CD161^hi^CD8^+^ T cells. **a** Absolute counts (x10^9^/L blood) of CD8^+^ MAIT cells. Mean ± SEM shown. On graph: *p = 0.0184, **p = 0.0026, ***p = 0.0002. **b** CD8^+^ MAIT cells as a percentage of live cells. Mean ± SEM shown. On graph: **p = 0.0040, *p = 0.0197, ***p = 0.0003. In (**a**, **b**), two-way mixed-effects models with Tukey’s multiple comparisons test after log transformation. For T1, T2, T3 and T4 respectively: SN (*n* = 11, *n* = 11, *n* = 10, *n* = 11), SP-NR (*n* = 5 for all), LR (*n* = 5, *n* = 6, *n* = 5, *n* = 5), HR (*n* = 9, *n* = 12, *n* = 12, *n* = 13). **c** Two-tailed Spearman correlations at T2 and T4 between CD8^+^ MAIT cells (x10^9^/L) in patients with HCMV reactivation (LR (green), HR (purple)) and the log_10_ area under the curve of HCMV DNA copies/mL over 0-100 days post-HSCT (AUC_0-100_). At T2, LR *n* = 6, HR *n* = 12. At T4, LR *n* = 5, HR *n* = 13. **d** Two-tailed Spearman correlations at T2 and T4 between CD8^+^ MAIT cell percentages in patients with HCMV reactivation (LR (green), HR (purple)) and the log_10_ HCMV DNA AUC_0-100_. **e** Median signal intensity (MSI) of CD38 on CD8^+^ MAIT cells. Mean ± SEM, two-way mixed-effects model with Tukey’s multiple comparisons test. For LR group, *p = 0.0105 between T2 and T4. At T4, **p = 0.006 for LR vs. HR; ^†^*p* = 0.0188 SN vs. LR; ^#^*p* = 0.0302 SP-NR vs. HR. For T1, T2, T3 and T4 respectively: SN (*n* = 9, *n* = 11, *n* = 10, *n* = 11), SP-NR (*n* = 4, *n* = 5, *n* = 5, *n* = 5), LR (*n* = 5, *n* = 6, *n* = 5, *n* = 5), HR (*n* = 7, *n* = 10, *n* = 12, *n* = 13). **f**, **g** Receiver-operating characteristic (ROC) curves for the performance of CD8^+^ MAIT cells (red), CD8^+^ T cells (blue) and CD4^+^ T cells (green) at T2 in discriminating LR and HR patients; (**f**) Shows absolute counts (x10^9^/L), (**g**) as percentages of live cells. Optimal CD8^+^ MAIT cell cut-offs were (**f**) 0.001337 × 10^9^ cells/L (91.67% sensitivity, 83.33% specificity) and (**g**) 0.0605% of live cells (100% sensitivity, 83.33% specificity). T1 is prior to detection; T2, at initial detection; T3, the peak; T4, near resolution of reactivation. SN HCMV-seronegative, SP-NR Seropositive no reactivation, LR Low-level reactivation, HR High-level reactivation. Source data are provided as a Source Data file.

MAIT cells are unconventional innate-like T cells which are abundant in the liver, gut and blood in healthy individuals, and are restricted by the major histocompatibility complex (MHC) class I-related molecule MR1^32,33^. We found an inverse relationship between the magnitude of HCMV DNAemia and circulating MAIT cell frequency in HSCT patients with HCMV reactivation (Fig. 5). Initial analysis focused on CD8^+^ MAIT cells (Vα7.2^+^CD161^high^ CD8^+^ T cells). CD8^+^ MAIT cell levels did not increase over time in any patient group, in line with reports of limited MAIT cell recovery in the first 1-2 years post-transplant^34,35^. However, CD8^+^ MAIT cell absolute counts (Fig. 5a) and percentages (Fig. 5B) were consistently higher in LR than HR across the time-points. Importantly, CD8^+^ MAIT cell levels were significantly higher in LR than HR at the initial detection of HCMV reactivation (T2) (Fig. 5a, b), a time-point at which the magnitude of HCMV DNAemia (Fig. 1c) and level of antigen exposure (HCMV AUC) were equivalent in LR and HR patients (Supplementary Fig. 1C; Supplementary Table 1), and which was before (median 9 (26-7) days) any administration of pre-emptive antiviral therapy.

The median percentage of CD8^+^ MAIT cells at T2 was 40.3-fold higher in LR than HR patients (medians 0.235% and 0.006% of live cells, respectively). CD8^+^ MAIT cell absolute counts and percentages were also significantly higher at T4 in LR compared to both HR and SN (Fig. 5a, b). To further add further context to the relevance of MAIT cells, similar findings were observed when the total MAIT cell population (CD3^+^Vα7.2^+^CD161^high^) was examined without prior gating on CD8 (Supplementary Fig. 6A, B), and when MAIT frequencies were measured as a percentage of total CD3^+^ cells (Supplementary Fig. 6C, D).

CD8^+^ MAIT cell frequencies at T2 and T4 in patients with HCMV reactivation inversely correlated with the HCMV DNA AUC_0-100_ (Fig. 5c, d) and peak HCMV copies/mL (Supplementary Fig. 7). Expression of the activation marker CD38 increased on CD8^+^ MAIT cells in HR, but not LR, at T3 and was significantly higher at T4 in HR compared to LR and SP-NR (Fig. 5e).

We generated receiver-operating characteristic (ROC) curves to estimate the ability of CD8^+^ MAIT cell frequencies at T2 to discriminate patients who proceeded to develop low- or high-level HCMV reactivation (Fig. 5f, g). Both CD8^+^ MAIT cell absolute counts (Fig. 5f) and percentages of live cells (Fig. 5g) demonstrated high AUCs of 0.89 (95%CI: 0.702-1.0; *p* = 0.0087) and 0.94 (95%CI: 0.828-1.0; *p* = 0.0027) respectively. CD8^+^ MAIT cells showed higher predictive ability than total CD8^+^ T cells or total CD4^+^ T cells (Fig. 5f, g), which are parameters assessed in routine post-transplant monitoring. The highest combined sensitivity (100%) and specificity (83.33%) was achieved using a threshold value of 0.0605% CD8^+^ MAIT cells (% of live cells). 12/13 patients with <0.0605% CD8^+^ MAIT cells at T2 ultimately developed high-level HCMV reactivation (12 HR; 1 LR), while all (5/5) patients above this cut-off experienced low-level HCMV reactivation. It is becoming increasingly apparent that changes in MAIT cell levels are associated with a number of viral infections^36–41^, and our data propose measurement of MAIT cell levels at the initial detection of HCMV DNAemia may serve as a potential marker for predicting the subsequent magnitude of HCMV DNAemia and anticipating the need for antiviral therapy.

### Expansion of effector-memory CD4^+^ T cells in HSCT recipients with low-level HCMV reactivation

**Fig. 6:**
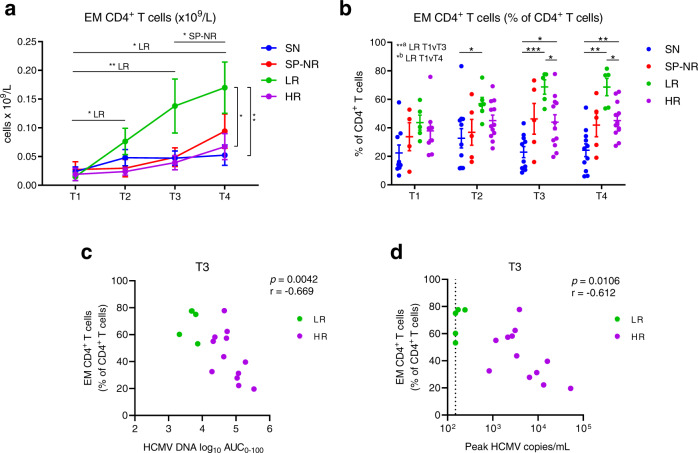
Expansion of effector-memory CD4^+^ T cells in HSCT recipients with low-level HCMV reactivation. **a** Absolute counts (x10^9^/L blood) of effector-memory (EM; CD45RA^-^CD45RO^+^CCR7^-^CD27^-^) CD4^+^ T cells. Mean ± SEM, two-way mixed-effects model with Tukey’s multiple comparisons test after log transformation. Horizontal lines display significant comparisons between indicated time-points for the patient groups shown: for LR, *T1-T2 *p* = 0.0213, **T1-T3 *p* = 0.0045, *T1-T4 *p* = 0.0447; for SP-NR, *T3-T4 *p* = 0.0428. Vertical brackets indicate significant differences between patient groups at T4 (*p = 0.0257 for LR vs. HR; **p = 0.0089 LR vs. SN). For T1, T2, T3 and T4 respectively: SN (*n* = 11, *n* = 11, *n* = 10, *n* = 11), SP-NR (*n* = 5 for all), LR (*n* = 5, *n* = 6, n = 5, *n* = 5), HR (*n* = 9, *n* = 12, *n* = 12, *n* = 13). **b** Percentage of CD4^+^ T cells with EM phenotype. Lines indicate mean ± SEM. Two-way mixed-effects model with Tukey’s multiple comparisons test. At T2, *p = 0.0399. At T3, *p = 0.0216 for SN vs. HR, *p = 0.0232 LR vs. HR, ***p = 0.0003 SN vs. LR. At T4, *p = 0.0483 LR vs. HR, **p = 0.0071 SN vs. HR, **p = 0.0013 SN vs. LR. For LR; ^a^T1-T3 *p* = 0.0031, ^b^T1-T4 *p* = 0.0178. For T1, T2, T3 and T4 respectively: SN (*n* = 9, *n* = 11, *n* = 10, *n* = 11), SP-NR (*n* = 4, *n* = 5, *n* = 5, *n* = 5), LR (*n* = 5, *n* = 6, *n* = 5, *n* = 5), HR (*n* = 8, *n* = 12, *n* = 12, *n* = 13). **c** Two-tailed Spearman correlation between log_10_ area under the curve (AUC) of HCMV DNA copies/mL over 0–100 days post-transplant, and EM CD4^+^ T cell percentages (of total CD4^+^ T cells) at T3 (peak of HCMV reactivation) in HSCT recipients with HCMV reactivation (low-level (<250 copies/mL) reactivation (green; *n* = 5); high-level (>830 copies/mL) reactivation (purple; *n* = 12)). **d** Two-tailed Spearman correlation between peak HCMV copies/mL and EM CD4^+^ T cell percentages at T3. The vertical black dotted line at 150 HCMV copies/mL indicates the lower limit of quantitation of the HCMV DNA quantitative PCR assay. T1 is prior to detection of HCMV reactivation; T2, at initial detection; T3, the peak; T4, near resolution of HCMV reactivation. SN HCMV-seronegative (blue), SP-NR Seropositive no reactivation (red), LR Low-level HCMV reactivation (green), HR High-level HCMV reactivation (purple). Source data are provided as a Source Data file.

The recovery of EM CD4^+^ T cells was found to be faster in LR patients compared to HR patients (Fig. 6a, b). EM CD4^+^ T cells expanded more rapidly in the LR group over the course of reactivation, as reflected in absolute counts (Fig. 6a) and percentages within the CD4^+^ T cell compartment (Fig. 6b). There was no significant difference in EM CD4^+^ T cell levels between the patient groups before the detection of HCMV reactivation (T1). Recovery of EM CD4^+^ T cell counts in HR patients remained low and was similar to non-reactivators (Fig. 6a). Significantly higher EM CD4^+^ T cell percentages at T3 and T4 were seen in LR compared to HR (medians 75.0% vs. 41.6% at T3, and 76.9% vs. 42.5% at T4, for LR and HR respectively) (Fig. 6b). The percentage of EM CD4^+^ T cells at T3 inversely correlated with the HCMV AUC_0-100_ (Fig. 6c) and peak HCMV copy number (Fig. 6d) in reactivators.

## Discussion

This study details the evolution of changes in immune reconstitution at different phases of HCMV reactivation post-HSCT. We identified an inverse relationship between the magnitude of HCMV DNAemia and MAIT cell frequency in patients with HCMV reactivation, indicating MAIT cells may be a marker to determine which patients may benefit from early intervention. Stronger EM CD4^+^ T cell reconstitution was also evident in patients who experienced low-level HCMV reactivation, consistent with a role in viral control.

A major obstacle in management of HCMV reactivation post-HSCT is potential antiviral drug-related toxicities from ganciclovir and foscarnet. Prophylaxis with safer agents such as Letermovir still carries a risk of viral resistance^42^, expense and the potential for delayed HCMV-specific T cell recovery^43^. Early discrimination of low-level and high-level reactivators could facilitate the tailoring of therapeutic decisions, as patients who develop self-limiting, low-level HCMV DNAemia may benefit from a shortened duration of prophylaxis or higher viral load threshold for pre-emptive therapy initiation.

The viral load at initial detection of HCMV reactivation has limited ability to predict which patients will develop clinically-significant infection^44,45^. Building on work by us and others demonstrating the utility of mass cytometry to describe immune profiles associated with post-HSCT complications^46–51^, here we stratified patients by HCMV-serostatus and the magnitude of peak HCMV DNAemia, directly analysing time-points aligning with HCMV viral load kinetics, including before and at initial detection of HCMV DNAemia.

A critical finding was that MAIT cell levels at the initial detection of HCMV DNAemia (cut-off 0.0605% of live cells) could effectively discriminate patients who eventually developed high-level HCMV DNAemia (>830 peak copies/mL) and required antiviral pharmacotherapy, versus those with low-level HCMV DNAemia (<250 peak copies/mL) who almost all cleared the infection spontaneously. MAIT cells are innate-like T cells with a semi-invariant T cell receptor (TCR) (Vα7.2) and recognise bacterial- and fungal-derived riboflavin metabolites presented on the non-classical class I-related MHC molecule (MR1)^52^. MAIT cell levels were significantly lower in patients with high-level HCMV reactivation compared to low-level reactivation. To our knowledge, previous studies have not identified an association between MAIT cell recovery and infectious complications after allogeneic HSCT, including HCMV^35,53^. Reduced circulating MAIT cell frequencies have been observed in HCMV-seropositive healthy individuals^54^ and depletion of circulating MAIT cells has been reported in several other viral infections, linked to chronic immune activation^36–41,55–59^. Lower circulating MAIT cell frequencies have also been reported in severe GvHD^34,48,60–62^. In light of our findings, validation studies in a larger cohort will be important to evaluate the predictive ability of MAIT cell levels at the initial detection of HCMV reactivation in stratifying patients who subsequently develop low-level versus high-level HCMV reactivation. A multi-center prospective study employing targeted measurement of MAIT cells by flow cytometry may also allow the impacts of additional variables, such as age, sex, aGvHD, TCD, immunosuppressive therapies and any other co-infections, to be integrated into the analysis, which may aid in identifying specific MAIT cell threshold levels that best distinguish low- and high-level HCMV reactivators in different patient subgroups.

The functional role of MAIT cells in HCMV infection, if any, remains to be determined. There is currently no known viral ligand for MR1 and it is unknown if MAIT cells can directly recognize HCMV-infected cells. Interestingly, HCMV has been reported to encode mechanisms to downregulate MR1 in vitro^63^, suggesting the virus might benefit from evasion of TCR-dependent MAIT cell activation. In viral infections, MAIT cells can be activated in a TCR-independent manner through inflammatory cytokines such as IL-12, IL-18, and IL-15^37,64,65^. Cytokine-mediated activation of MAIT cells can elicit the production of cytotoxic granules and cytokines, such as IFN-γ and TNF, which may be a pathway by which MAIT cells contribute to antiviral responses^37,66^.

The development of an inflammatory cytokine environment in high-level reactivators might be hypothesised to contribute to the increased MAIT cell activation (as measured by CD38 expression) observed in HSCT patients with high-level HCMV DNAemia in this study. Previous studies have identified highly activated MAIT cell phenotypes in conjunction with reduced circulating MAIT cell numbers in patients with viral infections such as dengue virus, HIV, and SARS-CoV2^36–41^. MAIT cell function is often impaired in virally-infected individuals^38,39,41,55^ and the functional recovery of MAIT cells in response to bacterial stimulation is reported to be slow after HSCT^35^. It will be of interest in future to evaluate the functional properties of MAIT cells in HSCT recipients with high- and low-level HCMV DNAemia.

The mechanisms underlying the difference in MAIT cell frequencies between low-level and high-level HCMV reactivators also require further investigation. Early MAIT cell recovery post-HSCT is partly driven by the proliferation of graft-derived MAIT cells^34^, and higher gut microbial diversity^60^ and abundance of Blautia spp. and Bifidobacterium longum^34^ have also been associated with better post-transplant MAIT cell recovery. The interaction between gut microbiota and immune cell reconstitution post-HSCT is becoming recognised^67–70^, with low intestinal microbial diversity connected with increased mortality and inferior clinical outcomes^71^. It is possible that poor MAIT cell recovery may be a biomarker of inferior overall immune reconstitution which predisposes to the development of high-level HCMV DNAemia.

MAIT cells exhibit tissue-homing properties^32^, thus recruitment of blood MAIT cells to tissue sites of inflammation^61^ might contribute to the lower frequency of circulating MAIT cells observed in high-level reactivators. Against this, Youssef, et al.^53^ were unable to detect TCR-Vα7.2^+^ lymphocytes in intestinal biopsies from paediatric HSCT recipients with aGvHD, HCMV reactivation or other viral infections. Examination of MAIT cells in HCMV-affected tissue biopsies in further studies represents an important avenue of research. Conditioning regimen intensity, thymic function and immunosuppressive therapies such as post-HSCT cyclophosphamide and cyclosporine A^34,35^ are among other factors which may influence MAIT cell recovery. In our cohort, two patients in the HR group received conditioning with post-transplant cyclophosphamide (those with haploidentical donors) and there was a greater proportion of patients with reduced-intensity conditioning and grade II aGvHD in the HR group.

Our data also pointed to faster EM CD4^+^ T cell recovery over the course of HCMV reactivation as a key feature distinguishing low-level reactivators from high-level reactivators. This supports the importance of CD4^+^ T cell responses in the control of HCMV reactivation^16,72–78^. Early HCMV-specific EM CD4^+^ T cell recovery post-HSCT has been associated with protection from HCMV reactivation^79^. HCMV-specific CD4^+^ T cell recovery is needed for effective HCMV-specific CD8^+^ T cell responses and HCMV clearance^80–82^. In the context of our current findings, EM CD4^+^ T cell expansion appears favourable for HCMV control. However, it is not yet known whether this is the key cell type whose reconstitution profile directly limits high level viraemia, or whether it is associated with such an outcome but is not itself the key effector cell type. Future mechanistic studies in the context of HSCT and HCMV infection (perhaps in animal models where such CD4^+^ T cells can be manipulated) will be an important component of work to determine the role of these cells in the context of preventing high level viraemia.

No significant differences in immune cell proportions were identified when directly comparing reactivators and non-reactivators before the detection of reactivation (T1; median day 19 post-HSCT), although a small number of differences in absolute CD4^+^ T cell subsets counts were detected. At this early time-point, there is likely to be substantial heterogeneity in immune recovery due to the impacts of transplant conditioning and differences in graft composition. Assessment of HCMV-specific CD8^+^ T cell functional profiles is a promising predictive method for distinguishing spontaneous controllers from those who progress to clinically-significant HCMV reactivation^13,17^. Limitations in cell numbers and blood volume in the pre-engraftment period often restrict analysis of PBMCs collected before the detection of HCMV reactivation, but here we utilised CyTOF to maximise the data extracted from such samples, which could in future help to refine immunological prediction of HCMV reactivation early post-transplant. We observed high proportions of GzmB-expressing CD8^+^ T cells prior to the detection of HCMV DNAemia in a subset of patients, which might reflect an early HCMV-specific T cell response to HCMV reactivation below the limit of detection.

Our observations including memory skewing, accumulation of late-differentiated T cells and inversion of the CD4:CD8 ratio are features previously reported to be associated with HCMV reactivation post-HSCT^23,83–85^, and are consistent with a prominent impact of HCMV reactivation on T cell reconstitution. The activated immune environment seen at the initial detection of HCMV DNAemia may reflect an early response to HCMV replication and/or it may provide an immune environment that favours HCMV reactivation^86^. Elevated frequencies of GzmB^+^ γδ T cells and NKG2C^+^CD57^+^ NK cells emerged with the progression of HCMV reactivation and have previously been implicated in HCMV control post-HSCT^80,87–91^. A salient observation at T4 was significantly lower frequencies of naïve T cells and CD27^+^ T cells in reactivators compared to non-reactivators. Suessmuth, et al.^23^ previously described a contraction of the naïve T cell compartment in HSCT patients with HCMV reactivation, and linked the clonal expansion of HCMV-specific EM CD8^+^ T cells to lower TCRβ diversity and defects in the TCRβ repertoire, suggestive of possible thymic impairment.

Although accelerated CD8^+^ T cell recovery is a canonical feature associated with HCMV reactivation post-HSCT^23,73,83,84^, we found that only a subgroup of reactivators developed a numerically elevated CD8^+^ T cell dominated immune profile. This ‘CD8^high^’ signature was seen in a majority of low-level reactivators, and indeed LR patients displayed the fastest overall CD8^+^ T cell recovery, suggesting this well-described observation is associated with viral control. The presence of HCMV-specific CD8^+^ T cells in seropositive recipients is reported to correlate with faster total CD8^+^ T cell reconstitution during the first year post-HSCT^92^ and it will be informative to examine the antigen-specificity and function of the T cell subsets within this elevated quantitative profile. We previously found that development of an adaptive immune signature containing increased numbers of activated CD8^+^ T cells was associated with HCMV clearance in HSCT patients who received adoptive virus-specific T cell therapy for pharmaco-refractory HCMV reactivation^46^.

There were some differences in the male:female ratio between the groups in this study, most notably in the SN group (Table 1). Participants were accrued sequentially and reflect the patients presenting for treatment. Our study was not powered to identify sex differences in immune response which should be addressed in a larger study cohort. Future studies could also integrate assessment of HCMV viral strain diversity, as multiple strain infections in HSCT recipients with HCMV infection are common and have been linked to higher viral loads and poorer outcomes in some, but not all, studies^93–96^.

In conclusion, this study provides comprehensive insight into the evolution of immune reconstitution profiles at different phases of HCMV reactivation post-HSCT, with simultaneous examination of multiple innate and adaptive populations, encompassing both absolute cell counts and percentages, providing a nuanced perspective on patterns of immune reconstitution. Our findings suggest that MAIT cell levels at the initial detection of HCMV DNAemia could be further explored as a potential biomarker for anticipating the eventual magnitude of HCMV reactivation and requirement for antiviral therapy.

## Methods

### Study subjects

This study included 35 adults who underwent peripheral blood (*n* = 32) or bone marrow (*n* = 3) allogeneic HSCT at Westmead Hospital (Sydney, Australia) between 2015 and 2017. Patient demographic and transplant characteristics are outlined in Table 1. Patients with cryopreserved PBMC samples available at ≥2 time-points in the retrospective study-design (see ‘Study design’ below) were included. All patients gave written, informed consent in accordance with the Declaration of Helsinki. No patient compensation was provided. This study was approved by the University of Sydney and Western Sydney Local Health District ethics committees (Project Number 2014/440).

Transplant conditioning regimens were myeloablative conditioning with cyclophosphamide-busulfan (*n* = 11) or cyclophosphamide-total body irradiation (*n* = 3); or reduced intensity conditioning with fludarabine-melphalan (*n* = 16), fludarabine-cyclophosphamide (*n* = 3), or fludarabine-cyclophosphamide with total body irradiation (one fraction) (*n* = 2). Four patients with matched unrelated donors received Tocilizumab as a component of conditioning (*n* = 3 SN, *n* = 1 SP-NR). Twenty patients received in vivo T cell depletion with anti-thymocyte globulin (*n* = 17) or alemtuzumab (*n* = 3) (Table 1). Donor and/or recipient pre-transplant EBV serostatus was available for 34/35 patients; all (34/34) were donor and/or recipient EBV seropositive. GvHD prophylaxis was according to institutional protocols depending on the indication for transplant, donor source and conditioning regimen.

### Isolation of peripheral blood mononuclear cells

Peripheral blood was collected prospectively at weekly or greater intervals until day 100 post-HSCT, in EDTA vacutainers. Peripheral blood mononuclear cells (PBMCs) were isolated by Ficoll-Paque PLUS (GE Healthcare) density-gradient centrifugation, washed in DPBS (without Ca or Mg), and cryopreserved in freezing media (RPMI-1640 (with L-glutamine) supplemented with 20% (v/v) foetal bovine serum and 10% (v/v) dimethyl sulfoxide). Cells were stored at −80 ^o^C for up to 7 days, then transferred to vapour-phase liquid nitrogen until use.

### Virological monitoring

Patients underwent weekly monitoring for HCMV and Epstein-Barr virus (EBV) in the first 100 days post-transplant. HCMV DNA load was measured in EDTA plasma by quantitative PCR (COBAS® AmpliPrep/COBAS® TaqMan® CMV Test; Roche). HCMV reactivation (also referred to as DNAemia) was defined as any detectable HCMV DNA in plasma. The lower limit of quantitation (LLQ) was 150 HCMV DNA copies/mL. The conversion to international units (IU) is as follows: HCMV IU/ml = 0.91 x HCMV copies/ml. EBV reactivation was defined as detection of EBV DNA at any level of quantitation using the EBV ELITe MGB® Kit (Elitech, Australia) with the conversion to EBV IU/ml being 0.77 x EBV copies/ml. The linear range of the assay was 150 to 10,000,000 copies/mL.

For graphical and calculation purposes, detectable HCMV DNA levels below the LLQ ( < 150 copies/mL) were assigned the value of 150 copies/mL. ‘Not detected’ readings (undetectable viral load) were assigned the value of 0 copies/mL. One HCMV-seronegative (D-/R-) patient had detection of HCMV DNA below the LLQ at a single time-point; this was considered a false positive and the patient was not excluded. It is possible that plasma HCMV DNA detection in the SN group might arise from PBMC contamination or damage during plasma collection, as detection of HCMV DNA in PBMCs from a small proportion of HCMV seronegative individuals has been reported^97^.

The log_10_ area under the curve (AUC) of HCMV DNA copies/mL plasma was calculated in GraphPad Prism version 8.2.1 (GraphPad Software, LLC) using the trapezoid rule. The duration of HCMV reactivation was calculated by first day minus last day of detected HCMV DNAemia, with any non-consecutive HCMV DNAemia episodes, including those extending beyond the first 100 days post-transplant, added together.

### Antiviral therapy for HCMV reactivation

HCMV-seropositive recipients received ganciclovir or valganciclovir prophylaxis from day -10 until day -1 pre-HSCT. Post-transplant HCMV reactivation was managed with pre-emptive antiviral therapy based on quantitative HCMV DNAemia measured weekly on peripheral blood plasma. Post-transplant pre-emptive antiviral therapy with ganciclovir and/or foscarnet was administered to 14/19 patients with HCMV reactivation. Antiviral pharmacotherapy was commenced when 2 consecutive HCMV PCR results showed increasing copy numbers, if a single result was greater than 1000 copies/mL or according to physician discretion if a HCMV viraemic patient was considered to be at high risk of uncontrolled HCMV replication. Ganciclovir 5 mg/kg IV 2 times daily (or dose equivalent of oral valganciclovir) was the first line agent and Foscarnet 60 mg/kg 3 times daily was used if (val)ganciclovir was contraindicated. A primary course of antiviral pharmacotherapy lasted 14 days, after which further treatment depended on response.

The point of pre-emptive therapy initiation for each patient is indicated on the HCMV DNAemia graphs in Fig. 1a by the shift from the solid line (before therapy) to dotted line (after initiation of therapy). Of the 14 patients who received pre-emptive antiviral therapy for HCMV reactivation, 8/14 patients had received pre-emptive therapy at or before T3, and 14/14 patients had received pre-emptive therapy by T4. No patient received pre-emptive antiviral therapy at or before T2.

### Study design

The patients were retrospectively divided into four groups according to pre-transplant HCMV serostatus and magnitude of post-transplant HCMV reactivation (Fig. 1a). The ‘Seronegative’ (SN; *n* = 11) group were HCMV-seronegative recipients with seronegative donors (D-/R-), and had no detected HCMV DNAemia. The ‘Seropositive No Reactivation’ (SP-NR; *n* = 5) group were HCMV-seropositive recipient (R + ) and/or donor (D + ) patients with no documented HCMV reactivation in the first 100 days post-HSCT. ‘Low-level reactivators’ (LR; *n* = 6) developed HCMV reactivation to <250 peak copies/mL. ‘High-level reactivators’ (HR; *n* = 13) developed HCMV reactivation to >830 peak copies/mL. No patients had peak HCMV titres between 250 and 830 copies/mL.

Mass cytometry was performed on PBMC samples from four time-points retrospectively selected in relation to the course of HCMV DNAemia (Fig. 1b): (T1) prior to detection of HCMV DNAemia, (T2) at the initial detection of DNAemia, (T3) at the peak of HCMV DNAemia, and (T4) near the resolution of HCMV DNAemia (Supplementary Table 2). Samples from matched days post-transplant were analysed from HSCT recipients who did not experience HCMV reactivation (SN and SP-NR) (Fig. 1d).

A total of 130 PBMC samples were analysed. 9/35 patients (*n* = 5 HR, *n* = 3 LR, *n* = 1 SN) did not have samples available at all four time-points. Of these patients, 8/9 were missing a sample from a single time-point, and 1/9 was missing samples from two time-points. There was a median of 19 (5–49) days between consecutive time-point samples analysed from an individual patient.

If cryopreserved PBMCs were not available from the precise days of T2, T3, or T4 time-points, the most proximate samples (where available) were used. For T2, if a PBMC sample from the day of first detected HCMV DNAemia was not available, the immediately succeeding available sample was used. If the precise T3 sample was not available, the most proximate sample immediately preceding the HCMV DNAemia peak was used. For T4, the sample at or most proximate to HCMV DNAemia resolution (undetectable viral load) in first 100 days post-transplant was analysed. At T4, with the exception of 3 HR patients, HCMV DNAemia was undetectable (6/13 HR and 6/6 LR) or had declined below the LLQ (4/13 HR). For patients with unresolved HCMV DNAemia in the first 100 days post-HSCT (*n* = 7), T4 samples corresponded to suppression of HCMV DNAemia below the LLQ (*n* = 4) or otherwise matched days post-HSCT to the other HR patients (*n* = 3). The magnitude of HCMV DNAemia (in copies/mL plasma) at T4 in the patients with detectable HCMV DNAemia at T4 had decreased from peak levels by a median of 97.7% (range 83.2–98.8%).

### Mass cytometry staining, acquisition and analysis

#### Cell staining

PBMCs were stained for mass cytometry in eight batches using a panel of 36 metal-conjugated antibodies (Supplementary Table 3). All samples from an individual patient were included in the same staining batch. Each batch contained a representation of patient groups (SN, SP-NR, LR, HR) and a batch control PBMC sample from a healthy donor. All antibodies were validated, pre-titered and supplied in per-test amounts by the Ramaciotti Facility for Human Systems Biology Mass Cytometry Reagent Bank. Reagent Bank antibodies were purchased in a purified, carrier-free unlabelled format from commercial suppliers. Unlabelled antibodies were conjugated with the indicated metal isotope using the Maxpar Antibody Labelling Kit (Fluidigm, South San Francisco, CA) according to the manufacturer’s protocol. All antibodies were titrated before inclusion in the panel.

Briefly, cryopreserved PBMCs were quickly thawed in a 37 ^o^C waterbath and washed with warm RPMI-1640 (Lonza) supplemented with 10% (v/v) FBS (Sigma) and 1:10000 (v/v) Pierce Universal Cell Nuclease (Thermo Fisher Scientific). Cells were then washed with warm RPMI-1640 containing 10% FBS and counted using a haemocytometer and trypan blue. After one wash in serum-free RPMI-1640, up to 3.0 × 10^6^ viable cells per sample were retained for subsequent staining steps. For live/dead discrimination, cells were incubated with 1.25 μM Cell‐ID^TM^ cisplatin (Fluidigm) in serum-free RPMI-1640 for 3 min at room temperature (RT), followed by immediate quenching with RPMI-1640 containing 10% FBS. Cells were then washed once with FACS buffer (DPBS containing 1% FBS and 0.01 M EDTA) then incubated in a 50 μL surface antibody cocktail (prepared in FACS buffer) for 30 min at 4 ^o^C. The antibody cocktail mastermix was freshly prepared and centrifuged at 12,000 x g for 4 min through a 0.1 μm filter immediately before use. Following two washes with FACS buffer, cells were fixed and permeabilised by incubation in 1X FoxP3 Fixation/Permeabilisation buffer (eBioscience) for 45 min at 4 ^o^C, then washed twice with 1X Permeabilisation buffer (eBioscience). Cells were stained with a 50 μL intracellular antibody cocktail (mastermix prepared in 1X Permeabilisation buffer and centrifuged through a 0.1 μm filter immediately before use) for 45 min at 4^o^C. The cells were subsequently washed with 1X Permeabilisation buffer, then FACS buffer. Finally, cells were fixed in a 4% paraformaldehyde solution (prepared in PBS) containing 0.125 μM Cell-ID™ Iridium-191/193 nucleic acid intercalator (Fluidigm) for 20 min RT initially, then stored at 4 ^o^C overnight or for up to one week prior to acquisition.

#### Acquisition

On the day of acquisition, cells were washed once in FACS buffer, once in DPBS and then twice in MilliQ water. The pellet was resuspended at 0.8 × 10^6^ cells/mL in 1:10 (v/v) EQ Four Element Calibration Beads (Fluidigm) in MilliQ water, and filtered through a 0.35 μm nylon cell strainer snap-cap (Falcon) immediately prior to acquisition. Samples were acquired on CyTOF 2 Helios upgraded mass cytometer (Fluidigm) with an acquisition rate of up to 400 events/s and flow rate of 30 μL/min. A median of 311867 events were acquired per sample (*n* = 130). FCS files were normalised on the CyTOF Software (version 6.7.1014) using the signal intensity of the EQ beads acquired within each sample.

#### Analysis

Cell populations were identified via manual gating in FlowJo 10.0.7 (Tree Star, Inc.). The gating strategy for major subsets is outlined in Supplementary Fig. 2. A list of all cell subsets analysed is shown in Supplementary Table 4 and Supplementary Table 5. Analysis of cell subset percentages included both percent of live cells (% live) and/or percent of parent subset. Percentages of live cells (% live) were derived from the live cells gate indicated in Supplementary Fig. 2. Apart from major cell subsets, data were treated as missing if there were too few cells in the parent gate to allow for accurate gating of subpopulation(s). In these cases, the absolute counts were returned as zero.

Absolute cell counts (x10^9^/L blood) for each subset were calculated with reference to absolute monocyte (Mo) or lymphocyte (Ly) counts measured on a Sysmex 1800 full blood analyser on the day of PBMC sample collection. Briefly, the frequency of each cell subset as a percentage of total live cells was divided by either the sum of monocyte populations (classical, intermediate, non-classical) or the sum of lymphocyte populations (B cells, CD3^+^ cells, NK cells, mDC and pDC) as percentages of total live cells, and then multiplied by the absolute Mo or Ly count (x10^9^/L)^46^. Readings of ‘ND’ on the full blood analyser were assigned the value 0.5 × 10^8^ cells/L (half of the lowest quantified concentration for Ly and Mo). White blood cell (WBC) counts (x10^9^/L) were derived directly from the full blood analyser.

### Statistical Analysis

All statistical tests were performed using GraphPad Prism Software version 8.2.1 (GraphPad Software, LLC) unless indicated otherwise. To compare patient characteristics between groups, the Fisher’s exact test (categorical variables) or Mann-Whitney test (continuous variables) were used as appropriate. All tests were two-sided and comparisons with *p* < 0.05 were considered significant. Two-class unpaired significance analysis of microarrays (SAM)^98^ in MultiExperiment Viewer 4.9.0 (TM4)^99^ was used to compare cell-subset frequencies between pairs of patient groups. Cell proportions (88 subsets) and absolute counts (77 subsets) were z-score standardised and analysed independently for each time-point, using 100 permutations with delta adjusted to yield a false discovery (FDR) rate of zero. If no significant subsets were identified using this approach, delta was tuned to the next threshold and significant subsets with a FDR < 0.05 were reported if found. Delta values used were as follows: Fig. 3a: T1 (delta = 0.300), T2 (delta = 0.623), T3 (delta = 0.781), T4 (delta = 0.883). Figure 3b: T1 (delta = 0.293), T2 (delta = 0.487), T3 (delta = 0.577), T4 (delta = 0.571). Heat map columns represent individual patients and are preserved in the same order across heat maps, except where samples were unavailable per time-point.

To further examine significant cell subsets as identified by SAM, two way mixed-effects models with the Geisser-Greenhouse correction followed by Tukey’s multiple comparisons test were used to enable comparison of cell subset frequencies both (a) between the four patient groups at each of four time-points, and (b) between the four time-points for each patient group. The mixed effects model is similar to repeated measures ANOVA, but allows for missing values. The mixed model implemented in GraphPad Prism uses a compound symmetry covariance matrix and is fit using restricted maximum likelihood (REML). The absolute counts of immune cell subsets were observed to have positively skewed distributions, thus data were log_10_-transformed to satisfy the assumption of normality prior to fitting the mixed effects model. Residual and QQ normality plots were visualised to confirm suitability of the model on the log-normal distribution. All absolute cell count graphs display untransformed data, with significance evaluated using log-transformed values. Correlations were evaluated using two-tailed Spearman correlations. Receiver-operating characteristic (ROC) curves were generated in GraphPad Prism 8.3.1. Data stated in the main text is the median with range in brackets.

### Reporting summary

Further information on research design is available in the Nature Research Reporting Summary linked to this article.

## Supplementary information

Supplementary Information

Reporting Summary

## Data Availability

The data underlying all tables and figures are provided in the Source Data file. Other data generated during and/or analysed during the current study are available from the corresponding author (B.S.) on request. Source data are provided with this paper.

## Source data

Source Data
