# Supplementary material for: Immunoprofiling reveals cell subsets associated with the trajectory of cytomegalovirus reactivation post stem cell transplantation
Source: Nat Commun. 2022 May 11;13:2603. doi: 10.1038/s41467-022-29943-9 (PMC9095831; doi:10.1038/s41467-022-29943-9)
Supplement: Supplementary file 1 — Supplementary Information [file 41467_2022_29943_MOESM1_ESM.pdf]

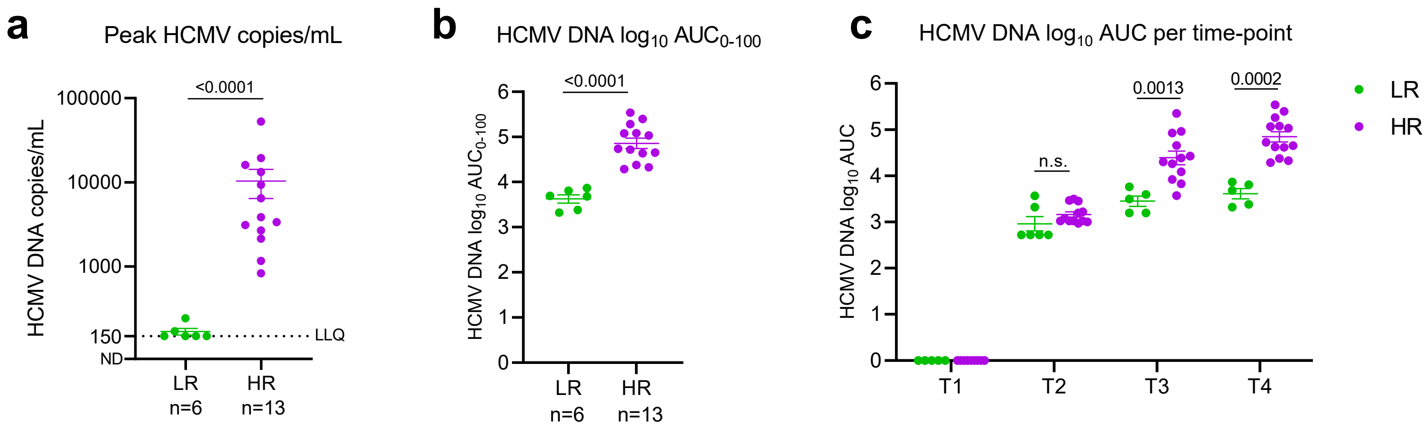

<u>Supplementary Figure 1.</u> Magnitude of HCMV reactivation in HSCT recipients.

(**a**) Peak HCMV copies/mL plasma in HSCT recipients with low-level (LR; green) or high-level (HR; purple) HCMV reactivation. The LLQ (lower limit of quantitation; 150 copies/mL) of the HCMV DNA quantitative PCR assay is indicated by the horizontal black dotted line. ND, not detected. Statistical significance between LR and HR evaluated using a two-tailed Mann-Whitney U test; p value indicated. (**b**) The HCMV DNA $\log_{10}$ area under the curve (AUC) in the first 100 days post-HSCT (AUC_{0-100}) in LR and HR patients. Statistical significance between LR and HR evaluated using a two-tailed Mann-Whitney U test; p value indicated. (**c**) The HCMV DNA $\log_{10}$ AUC in LR and HR patients at each time-point (T) analysed by mass cytometry. T1 (LR n=5, HR n=9), T2 (LR n=6, HR n=12), T3 (LR n=5, HR n=12), T4 (LR n=5, HR n=13). Statistical significance between LR and HR was evaluated per time-point using a two-tailed Mann-Whitney U test, $p<0.05$ considered significant. n.s., not significant. Graphs show mean ± SEM.

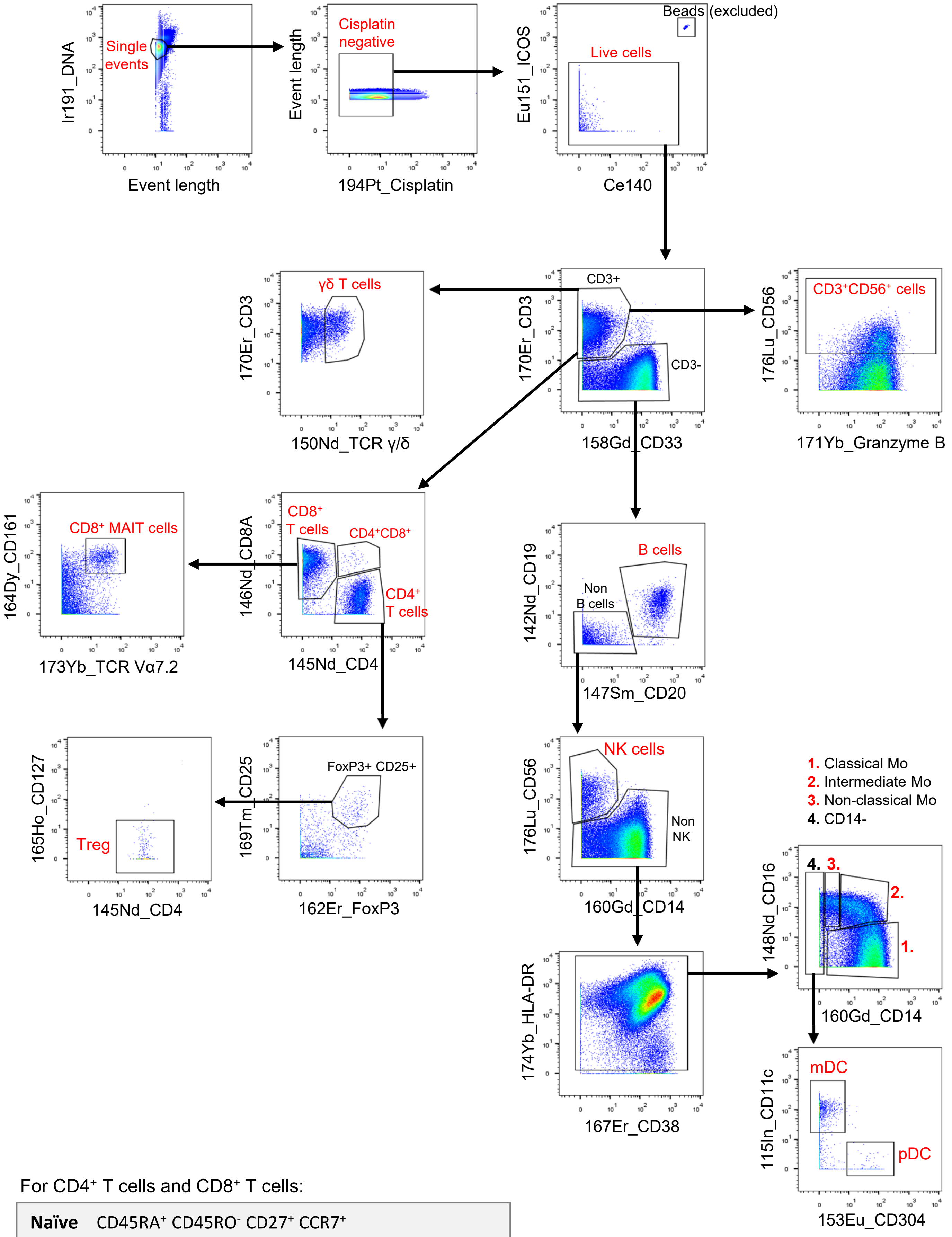

For $CD4^{+}$ T cells and $CD8^{+}$ T cells:

Naïve $CD45RA^{+}$ $CD45RO^{-}$ $CD27^{+}$ $CCR7^{+}$

Central memory (CM) $CD45RA^{-}$ $CD45RO^{+}$ $CD27^{+}$ $CCR7^{+}$

Effector memory (EM) $CD45RA^{-}$ $CD45RO^{+}$ $CD27^{-}$ $CCR7^{-}$

EMRA $CD45RA^{+}$ $CD45RO^{-}$ $CD27^{-}$ $CCR7^{-}$

Supplementary Figure 2. Mass cytometry gating strategy for major subsets.

<u>Supplementary Figure 2.</u> Mass cytometry gating strategy for major subsets.

Single events were distinguished from doublets and debris by DNA intercalator staining and event length. Cisplatin-negative (live) cells were then selected and EQ beads ($Eu151^{+}Ce140^{+}$) excluded prior to identification of major PBMC populations, as per the gating strategy shown. The 'live cells' gate indicated was the gate from which percentages of immune cell subsets (out of total live single cells) were derived. The phenotype used for identification of naïve and memory T cell subsets is shown (grey box). Further gating on cell subpopulations as listed in Supplementary Table 4 was conducted. All gating was performed in FlowJo software version 10.0.7 (Tree Star, Inc.). Mo, monocyte.

a Proportions

T2

- CD86$^+$ (% of classical mo)
- CD86$^+$ (% of intermediate mo)
- CD86$^+$ (% of mDCs)
- CD86$^+$ (% of non-classical mo)
- CD86$^+$ (% total mo)
- CD38$^+$ (% of CD8$^+$ MAIT cells)

T2, T3

- HLA-DR$^+$CD38$^+$ (% of EM CD8$^+$ T cells)
- ICOS$^+$ (% of CD4$^+$ T cells)
- ICOS$^+$ HLA-DR$^+$CD38$^+$ (% of CD4$^+$ T cells)

T3

- CM (% of CD8$^+$ T cells)
- HLA-DR$^+$CD38$^+$ EM (% of CD8$^+$ T cells)
- HLA-DR$^+$ (% of NK cells)

T3, T4

- CD38$^+$ (% of non-classical mo)
- CD38$^+$GzmB$^+$ (% of CM CD8$^+$ T cells)
- CD57$^+$GzmB$^+$ (% of CD8$^+$ T cells)
- EM (% of CD8$^+$ T cells)
- GzmB$^+$ (% of CD8$^+$ T cells)
- GzmB$^+$ (% of CD4$^+$ T cells)
- CD57$^+$ (% of CD4$^+$ T cells)
- CD57$^+$PD-1$^+$CD38$^+$ (% of CD4$^+$ T cells)
- GzmB$^+$ (% of γδ-T cells)

T4

- CD57$^+$NKG2C$^+$ (% of NK cells)
- CD57$^+$ (% of CD8$^+$ T cells)
- CD57$^+$ CD8$^+$ T cells (% of live cells)
- CD57$^+$CD38$^+$ (% of CD8$^+$ T cells)
- CD8$^+$ T cells (% of live cells)
- CD57$^+$GzmB$^+$ (% of CD3$^+$CD56$^+$ cells)
- CD3$^+$CD56$^+$ cells (% of CD3$^+$ cells)
- γδ-T cells (% of live cells)
- CD45RA$^+$GzmB$^+$CD27$^-$ (% of γδ-T cells)
- ↓ CD27$^+$ (% of γδ-T cells)
- ↓ CM (% of CD4$^+$ T cells)
- ↓ CD27$^+$ (% of CD4$^+$ T cells)
- ↓ CD27$^+$ (% of CD8$^+$ T cells)
- ↓ Naïve (% of CD8$^+$ T cells)

T2, T3, T4

- CD38+ (% of CD4+ T cells)
- PD-1+ (% of CD4+ T cells)
- HLA-DR+CD38+ (% of CD4+ T cells)
- EM (% of CD4+ T cells)
- HLA-DR+CD38+ EM (% of CD4+ T cells)
- HLA-DR+CD38+ (% of EM CD4+ T cells)
- HLA-DR$^+$CD38$^+$ (% of CD8$^+$ T cells)

b Absolute counts

T1

- ↓ CD38$^+$ CD4$^+$ T cells
- ↓ CD4$^+$ T cells
- ↓ HLA-DR$^+$CD38$^+$ CD4$^+$ T cells
- ↓ ICOS$^+$ CD4$^+$ T cells
- ↓ PD-1$^+$ CD4$^+$ T cells

T1, T2

- ↓ CM CD4$^+$ T cells

T2

- ↓ WBC

T3

- CD57$^+$PD-1$^+$CD38$^+$ CD4$^+$ T cells
- B cells
- CD38$^+$GzmB$^+$ CM CD8$^+$ T cells
- CD57$^+$CD38$^+$ CD8$^+$ T cells
- CD8$^+$ T cells
- EM CD8$^+$ T cells
- HLA-DR$^+$CD38$^+$ CD8$^+$ T cells
- HLA-DR$^+$CD38$^+$ EM CD8$^+$ T cells
- PD-1$^+$ CD8$^+$ T cells
- PD-1$^+$ EM CD8$^+$ T cells
- Tim-3$^+$ CD8$^+$ T cells
- CD57$^+$ CD8$^+$ T cells

T3, T4

- CD161$^{lo/-}$ Vα7.2$^+$ CD8$^+$ T cells
- GzmB$^+$ CD8$^+$ T cells
- CD57$^+$GzmB$^+$ CD8$^+$ T cells
- CM CD8$^+$ T cells
- GzmB$^+$ CD4$^+$ T cells
- γδ-T cells
- GzmB$^+$ γδ-T cells
- CD45RA$^+$ γδ-T cells
- CD45RA$^+$GzmB$^+$CD27$^-$ γδ-T cells
- CD3$^+$CD56$^+$ cells
- CD57$^+$GzmB$^+$ CD3$^+$CD56$^+$ cells

T4

- NKG2C$^+$ CD8$^+$ T cells
- CD57$^+$NKG2C$^+$ NK cells

T1, T2, T4

- ↓ CD27$^+$ CD4$^+$ T cells
- ↓ Naïve CD4$^+$ T cells

c Percents and Counts

T3

- CD38$^+$GzmB$^+$ CM CD8$^+$ T cells
- CM CD8$^+$ T cells
- EM CD8$^+$ T cells
- HLA-DR$^+$CD38$^+$ CD8$^+$ T cells
- HLA-DR$^+$CD38$^+$ EM CD8$^+$ T cells
- CD57$^+$PD-1$^+$CD38$^+$ CD4$^+$ T cells

T3, T4

- GzmB$^+$ CD8$^+$ T cells
- CD57$^+$GzmB$^+$ CD8$^+$ T cells
- GzmB$^+$ CD4$^+$ T cells
- GzmB$^+$ γδ-T cells

T4

- CD57$^+$NKG2C$^+$ NK cells
- CD45RA$^+$GzmB$^+$CD27$^-$ γδ-T cells
- γδ-T cells
- CD3$^+$CD56$^+$ cells
- CD57$^+$GzmB$^+$ CD3$^+$CD56$^+$ cells
- ↓ CD27$^+$ CD4$^+$ T cells

Key
- Monocyte/DC
- NK cell
- CD8$^+$ T cell
- CD4$^+$ T cell
- γδ-T cell
- CD3$^+$CD56$^+$ cell
- B cell

Supplementary Figure 3. Schematic representation of all cell subsets shown on heat maps in Figure 3.

__Supplementary Figure 3.__ **Schematic representation of all cell subsets shown on heat maps in Figure 3.** The heat map rows in Figure 3 show cell subsets which differed significantly in percentage (Figure 3A) or absolute count (Figure 3B) in reactivators (LR and HR) compared to non-reactivators (SP-NR and SN), as determined by significance analysis of microarrays (SAM). (**a**) Visual representation of all cell subsets shown on Figure 3A heat maps, and (**b**) visual representation of all cell subsets displayed on Figure 3B heat maps, with text coloured by cell type. Coloured vertical lines adjacent to cell subsets indicate the time-point(s) at which the cell subsets were significantly higher in HCMV reactivation patients (LR, HR), compared to patients without reactivation (SN, SP-NR). Blue downward arrows are adjacent to cell subsets that were significantly lower in reactivators compared to non-reactivators. (**c**) Cell subsets which differed significantly in both percentage (Figure 3A) and absolute count (Figure 3B) between reactivators and non-reactivators. T1 is prior to the detection of HCMV reactivation; T2, at the initial detection of HCMV reactivation; T3, the peak; T4, near the resolution of HCMV reactivation. SN, HCMV seronegative (n=11); SP-NR, seropositive no reactivation (n=5); LR, low-level HCMV reactivation (n=6); HR, high-level HCMV reactivation (n=13).

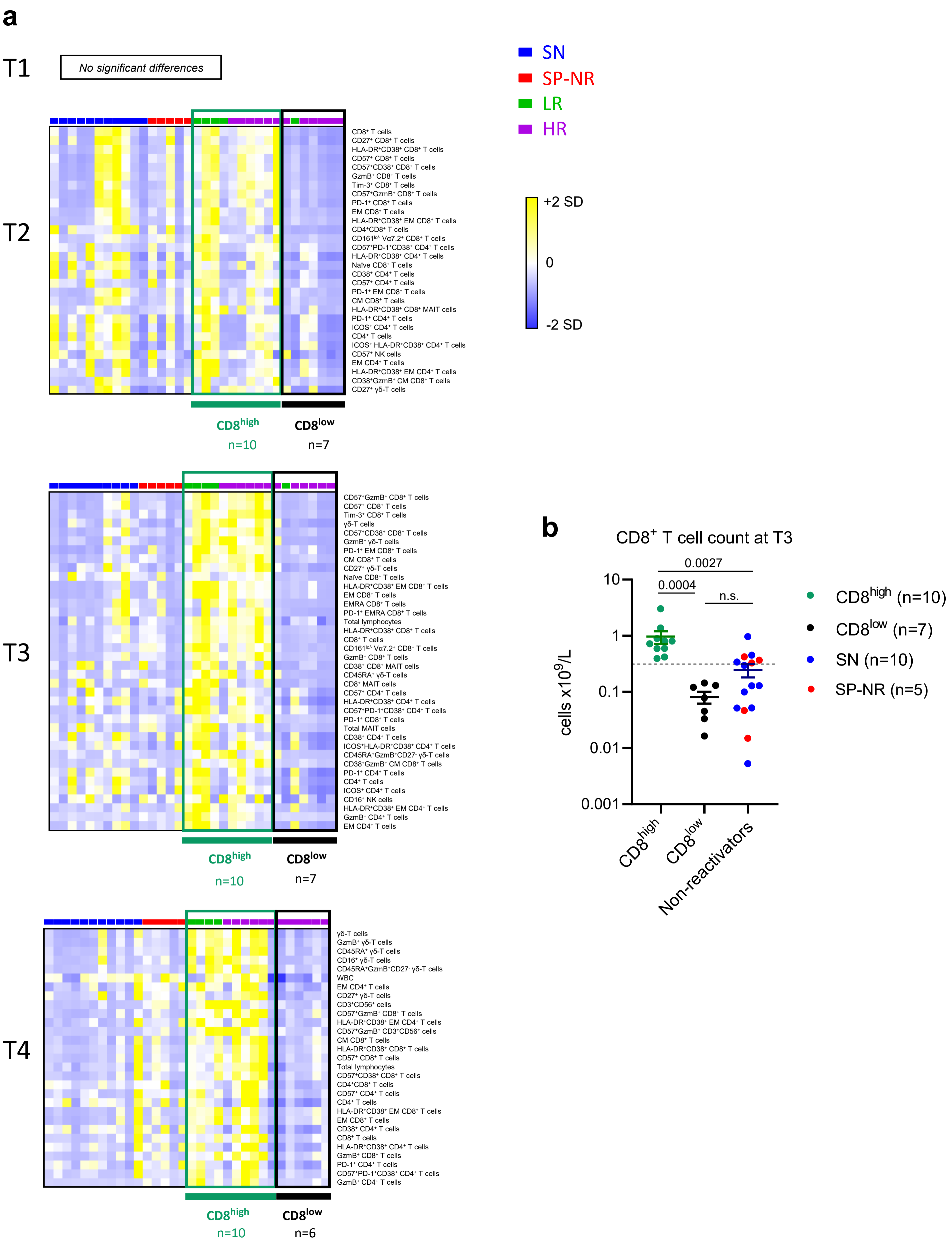

Supplementary Figure 4. **Elevated T cell signature in CD8^{high} reactivators compared to CD8^{low} reactivators.**

<u>Supplementary Figure 4.</u> Elevated T cell signature in CD8^{high} reactivators compared to CD8^{low} reactivators. HSCT recipients with HCMV reactivation were divided into 'CD8^{high}' (n=10) and 'CD8^{low}' (n=7) groups after detection of a CD8^{+} T cell dominated immune signature at T3 (peak of HCMV DNAemia) in Figure 3B in a subgroup of reactivators (termed 'CD8^{high}'). (**a**) Absolute counts of all (77) immune subsets were directly compared between CD8^{high} reactivators (dark green) and CD8^{low} reactivators (black) via two-class unpaired significance analysis of microarrays (SAM). Heat map rows display cell subsets that were significantly different in absolute count (x10^{9}/L blood) between CD8^{high} and CD8^{low} reactivators. As the CD8^{high} and CD8^{low} groups were defined at T3 (in Figure 3B), the two patients with HCMV reactivation who lacked a T3 sample (n=1 LR, n=1 HR) were not included in this analysis. Heat maps are coloured by the Z-score normalised per row. Each column represents a patient. SN (HCMV seronegative; blue) and SP-NR (seropositive no reactivation; red) patients are included on the heat maps for visual comparison. Delta values were as follows: T1 (delta=0.789), T2 (1.159), T3 (0.571), T4 (1.469). T1 is prior to the detection of HCMV reactivation; T2, at the initial detection of HCMV reactivation; T3, the peak; T4, near the resolution of HCMV reactivation. LR, low-level HCMV reactivation (light green); HR, high-level HCMV reactivation (purple); SD, standard deviation. (**b**) Absolute CD8^{+} T cell counts (x10^{9}/L blood) at T3 (the peak of HCMV DNAemia) in CD8^{high} reactivators (dark green), CD8^{low} reactivators (black), and non-reactivators (SN (blue) and SP-NR (red)). The black horizontal dotted line indicates the median CD8^{+} T cell count at T3 (0.3116 x10^{9}/L). Mean ± SEM is indicated. Statistical differences were determined by Kruskal-Wallis test with Dunn's multiple comparisons test; $p<0.05$ considered significant. n.s., not significant.

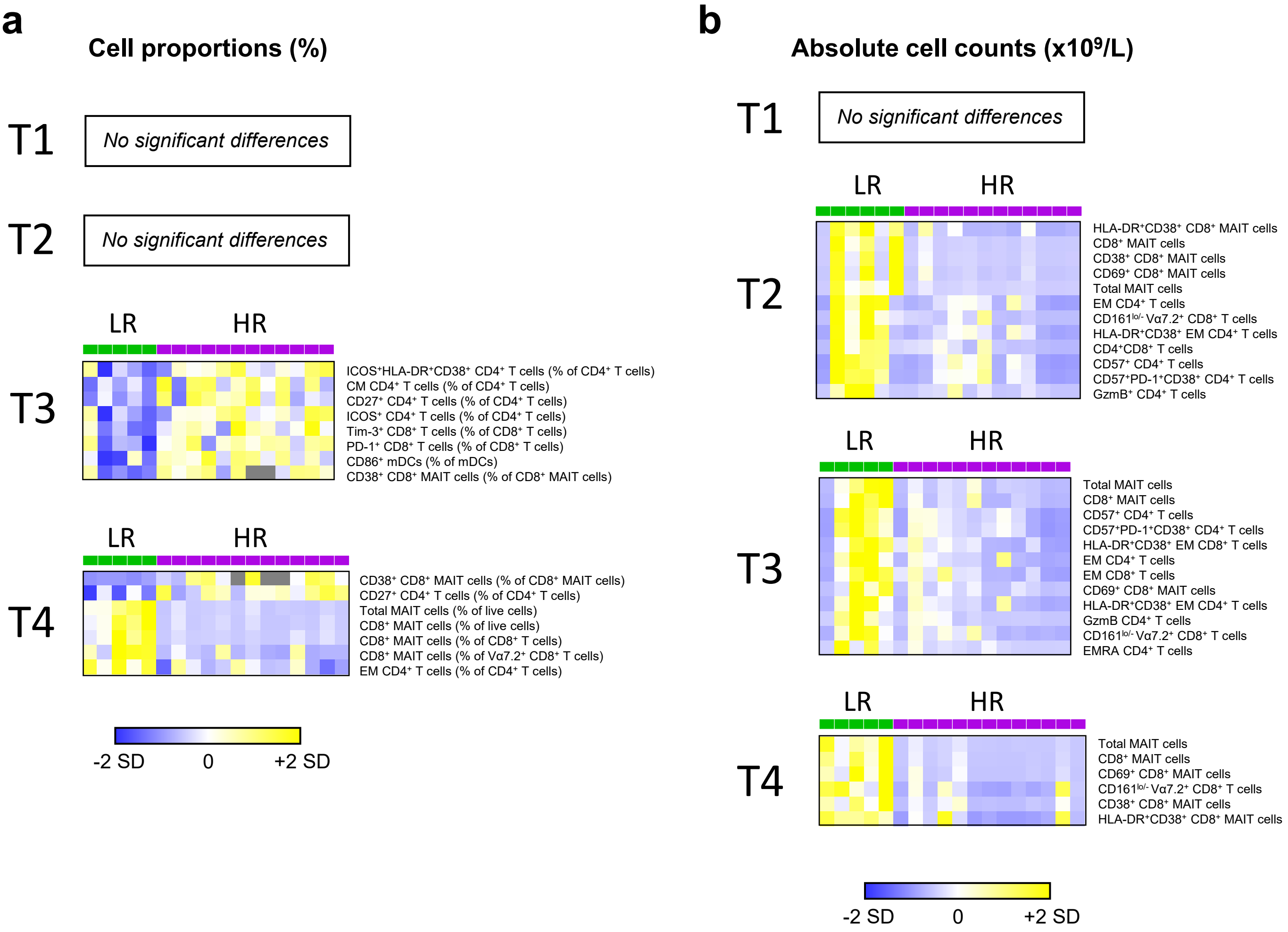

Supplementary Figure 5. Immune cell subset differences in HSCT recipients with low-level HCMV reactivation versus high-level HCMV reactivation.

Heat map rows display (**a**) percentages or (**b**) absolute counts ($x10^9$/L) of immune cell subsets that were significantly different between LR (green) and HR (purple) patients, as determined by two-class unpaired significance analysis of microarrays per time-point. Each column represents a patient. Heat maps are coloured by the Z-score normalised per row; grey colour indicates missing data (too few cells in parent gate). Delta values used were as follows: (a) T1 (delta=0.227), T2 (0.217), T3 (0.402), T4 (0.580); (b) T1 (delta=0.235), T2 (0.859), T3 (0.576), T4 (0.707). T1 is prior to the detection of HCMV DNAemia; T2 at initial detection of HCMV DNAemia; T3 is at the peak of HCMV DNAemia; T4, near the resolution of HCMV reactivation. LR, low-level HCMV reactivation; HR, high-level HCMV reactivation; SD, standard deviation. T2 (LR n=6, HR n=12), T3 (LR n=5, HR n=12), T4 (LR n=5, HR n=13).

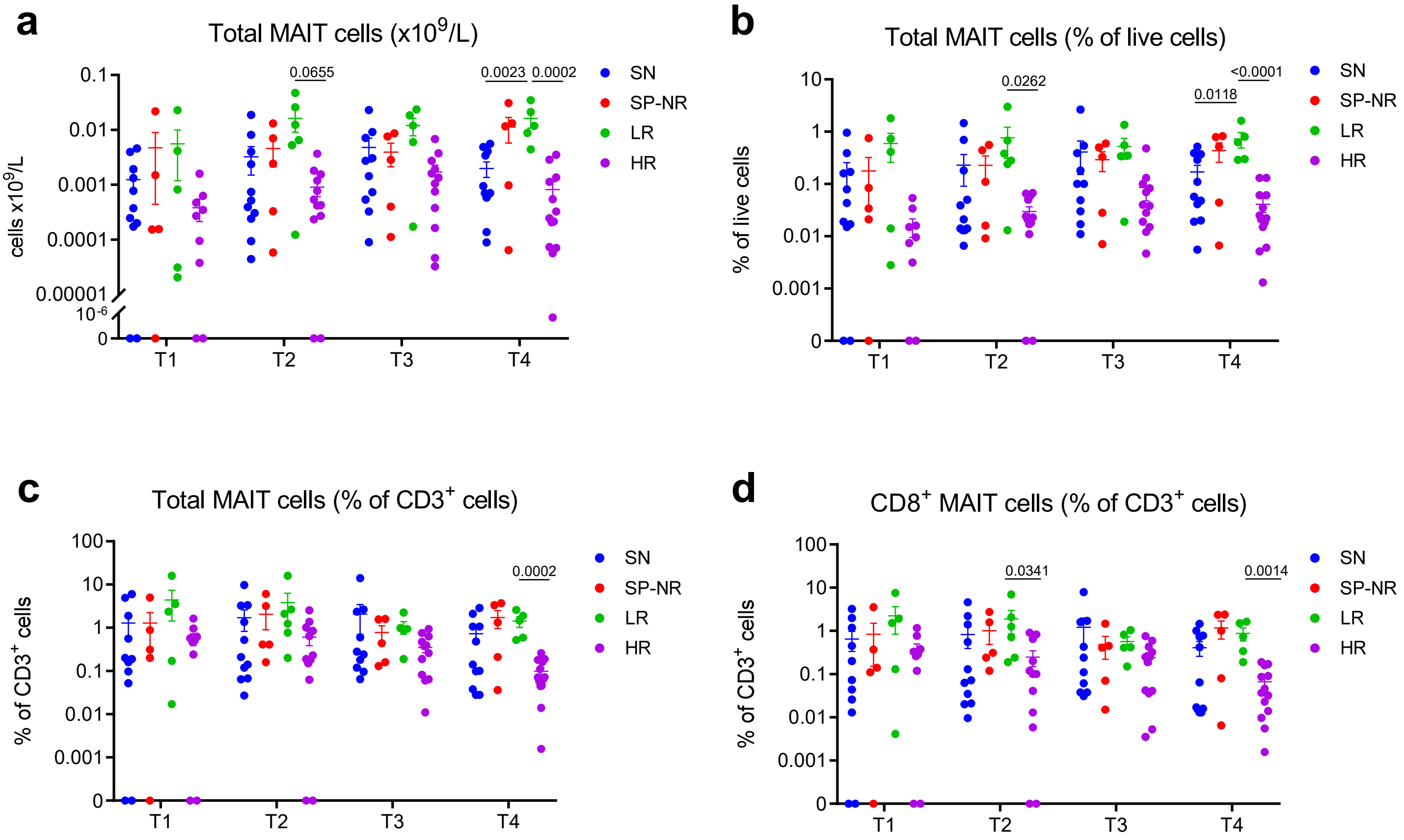

<u>Supplementary Figure 6.</u> Total MAIT cell ($CD3^+V\alpha7.2^+CD161^{hi}$) frequencies and MAIT cells as a percentage of total $CD3^+$ PBMCs. (**a**) Absolute counts ($x10^9/L$) of total MAIT cells ($CD3^+V\alpha7.2^+CD161^{hi}$). (**b**) Total MAIT cells ($CD3^+V\alpha7.2^+CD161^{hi}$) expressed as a percentage of total live PBMCs. (**c**) Total MAIT cells ($CD3^+V\alpha7.2^+CD161^{hi}$) expressed as a percentage of total $CD3^+$ cells. (**d**) $CD8^+$ MAIT cells ($V\alpha7.2^+CD161^{hi}$ $CD8^+$ T cells) expressed as a percentage of total $CD3^+$ cells. Mean ± SEM are indicated. Statistical significance between groups was assessed via two-way mixed-effects models with Tukey's multiple comparisons tests after log transform; $p<0.05$ was considered significant. SN, seronegative (blue); SP-NR, seropositive no reactivation (red); LR, low-level HCMV reactivation (green); HR, high-level HCMV reactivation (purple). T1 (SN n=11, SP-NR n=5, LR n=5, HR n=9), T2 (SN n=11, SP-NR n=5, LR n=6, HR n=12), T3 (SN n=10, SP-NR n=5, LR n=5, HR n=12), T4 (SN n=11, SP-NR n=5, LR n=5, HR n=13). T1 is prior to the detection of HCMV reactivation; T2, at the initial detection of HCMV reactivation; T3, the peak; T4, near the resolution of HCMV reactivation.

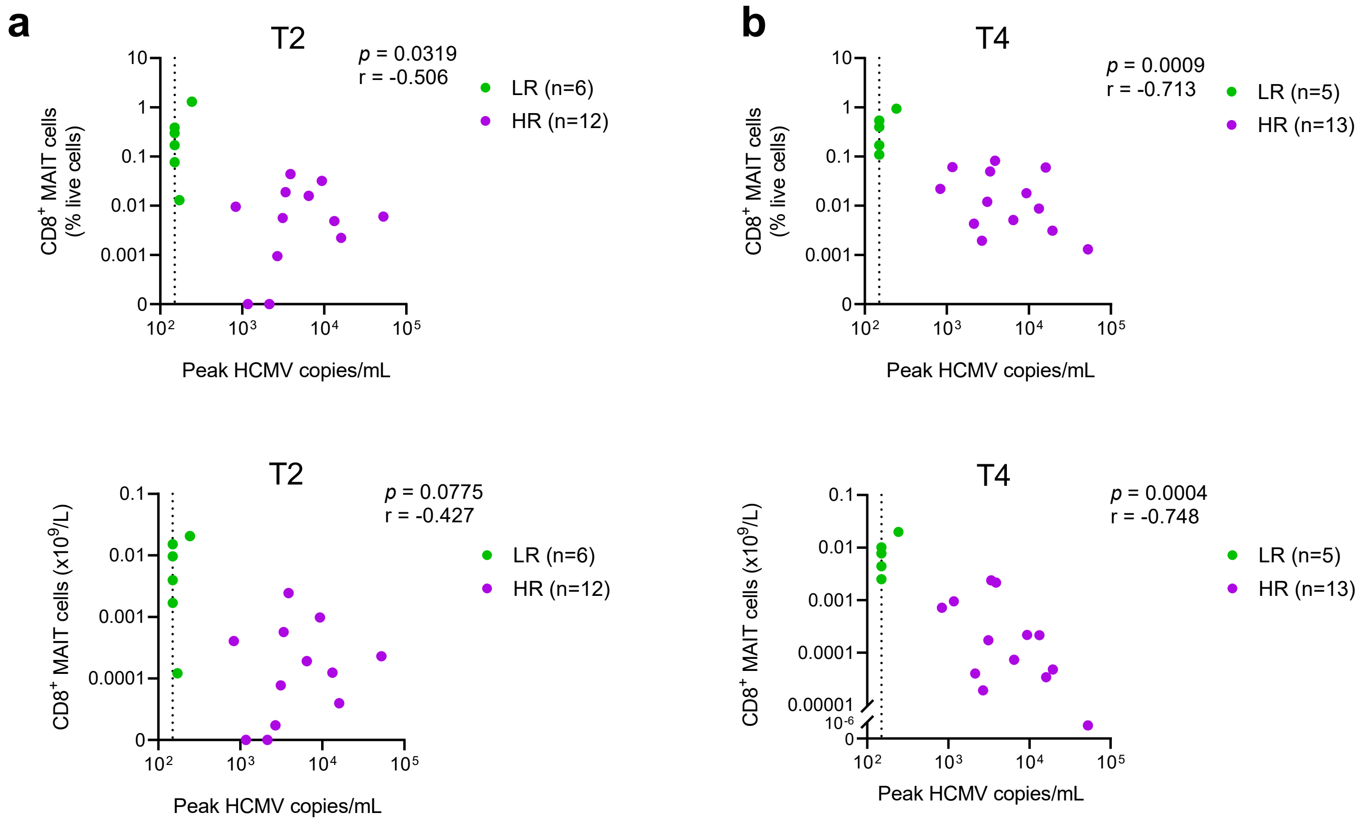

<u>Supplementary Figure 7.</u> Inverse correlation between peak HCMV copies/mL and CD8⁺ MAIT cell frequencies at T2 and T4. Two-tailed Spearman correlations between peak HCMV copies/mL and MAIT cell frequencies at (**a**) T2 (initial detection of HCMV DNAemia) and (**b**) T4 (near resolution of HCMV DNAemia) in HSCT recipients who experienced low-level HCMV reactivation (LR; green) and high-level HCMV reactivation (HR; purple). Correlations for CD8⁺ MAIT cell percentages (of live cells) and absolute counts ($x10^9$/L) are shown. $p<0.05$ was considered significant. The vertical black dotted line at 150 HCMV copies/mL plasma indicates the lower limit of quantitation (LLQ) of the HCMV DNA quantitative PCR assay.

<u>Supplementary Table 1.</u> HCMV DNAemia characteristics and antiviral treatment in patients with HCMV reactivation

	**All HCMV reactivators (n=19)**	**LR (n=6)**	**HR (n=13)**	**p value**
**Day post-HSCT of initial HCMV DNAemia detection**	29 (12-46)	35 (12-45)	28 (17-46)	0.6257
**Magnitude of first detected HCMV DNAemia (copies/mL)**	150 (150-515)	150 (150-150)	150 (150-515)	0.5170
**Peak HCMV DNA copies/mL**	2960 (150-52740)	150 (150-244)	3873 (836-52740)	**<0.0001**
**Duration of HCMV DNAemia (days)**	38.0 (7-121)	21.5 (7-49)	60.0 (28-121)	**0.0180**
**HCMV DNA log_{10} AUC**				
T2	3.02 (2.72-3.57)	2.72 (2.72-3.57)	3.05 (2.95-3.50)	0.2769
T3	4.09 (3.20-5.35)	3.50 (3.20-3.76)	4.35 (3.57-5.35)	**0.0013**
T4	4.62 (3.32-5.54)	3.68 (3.32-3.87)	4.73 (4.29-5.54)	**0.0002**
Total in first 100 days post-HSCT	4.64 (3.32-5.54)	3.69 (3.32-3.87)	4.74 (4.29-5.54)	**<0.0001**
**Antiviral therapy for HCMV reactivation (n patients)**	14 (74%)	1 (17%)	13 (100%)	**0.0005**

<u>Notes:</u> The days post-transplant to initial detection of HCMV reactivation were compared between LR and HR using a two-tailed unpaired t test. Two-tailed Mann-Whitney tests were used for other comparisons. p values indicate comparison between LR and HR groups; $p<0.05$ was considered significant (shown in bold font). AUC (area under the curve), LR (low-level HCMV reactivation), HR (high-level HCMV reactivation), HSCT (haematopoietic stem cell transplant), HCMV (human cytomegalovirus). HCMV AUC not shown for T1 (as T1 is prior to the detection of HCMV DNAemia). 'Antiviral therapy' refers to pre-emptive therapy.

<u>Supplementary Table 2.</u> Details of sample time-points for mass cytometry

	No HCMV reactivation		HCMV reactivation		
	SN (n=11)	SP-NR (n=5)	LR (n=6)	HR (n=13)	All patients (n=35)
**Number of samples (n)**					
T1	11	5	5	9	30
T2	11	5	6	12	34
T3	10	5	5	12	32
T4	11	5	5	13	34
**Days post HSCT**					
T1	19 (17-26)	20 (19-25)	19 (17-24)	18 (14-32)	19 (14-32)
T2	34 (33-41)	34 (32-42)	36 (31-45)	33 (27-48)	33.5 (27-48)
T3	50 (45-61)	47 (46-61)	47 (46-59)	46 (39-62)	47 (39-62)
T4	83 (75-89)	83 (82-89)	82 (67-87)	84 (74-89)	83 (67-89)
**Days from initial HCMV DNAemia**					All reactivation patients (n=19)
T1	n/a	n/a	-21 (-21 to -7)	-7 (-28 to -3)	-14 (-28 to -3)
T2	n/a	n/a	0 (0-21)	4.5 (0-19)	3 (0-21)
T3	n/a	n/a	21 (7-42)	21 (12-28)	21 (7-42)
T4	n/a	n/a	56 (22-70)	54 (37-69)	55 (22-70)
**Days from peak HCMV DNAemia**					
T1	n/a	n/a	-35 (-42 to -28)	-31 (-42 to -24)	-32.5 (-42 to -24)
T2	n/a	n/a	-17.5 (-28 to -7)	-16.5 (-23 to -8)	-16 (-28 to -7)
T3	n/a	n/a	0 (-14 to 0)	0 (-7 to 0)	0 (-14 to 0)
T4	n/a	n/a	28 (13-37)	35 (17-46)	31.5 (13-46)

<u>Notes:</u> Data are presented as absolute values (n) or median (range). SN (HCMV seronegative; D-/R-), SP-NR (HCMV seropositive no reactivation), LR (low-level HCMV reactivation; <250 peak HCMV DNA copies/mL), HR (high-level HCMV reactivation; 830-53,000 peak HCMV DNA copies/mL), HCMV (human cytomegalovirus), HSCT (haematopoietic stem cell transplant), n/a (not applicable). 'Samples' refers to PBMC samples.

<u>Supplementary Table 3</u>. Mass cytometry antibody panel

Metal	Specificity [anti-human]	Clone	Concentration (µg/mL)	Supplier	Catalogue number	Stain type
89Y	CDK1	Cdc2 p34 (17)	8	Santa Cruz Biotechnology	sc-54	Intracellular
115In	CD11c	Bu15	1	BioLegend	337202	Surface
141Pr	CD27	M-T271	1	Becton Dickinson	555439	Surface
142Nd	CD19	HIB19	1	Becton Dickinson	555410	Surface
143Nd	CD45RA	HI100	2	Becton Dickinson	555486	Surface
144Nd	CD69	FN50	3	BioLegend	310939	Surface
145Nd	CD4	RPA-T4	2	Becton Dickinson	555344	Surface
146Nd	CD8A	RPA-T8	8	BioLegend	301002	Surface
147Sm	CD20	2H7	4	BioLegend	302302	Surface
148Nd	CD16	3G8	1	Becton Dickinson	556617	Surface
149Sm	TIM3	7D3	1	Becton Dickinson	565768	Surface
150Nd	TCR γ/δ	B1	8	Becton Dickinson	555715	Surface
151Eu	CD278	DX29	4	Becton Dickinson	557801	Surface
152Sm	CD45RO	UCHL1	8	Becton Dickinson	555491	Surface
153Eu	CD304	12C2	1	BioLegend	354502	Surface
154Sm	CD163	GHI/61	6	Becton Dickinson	556017	Surface
155Gd	CD314 (NKG2D)	1D11	6	Becton Dickinson	552866	Surface
156Gd	CD86	IT2.2	2	Becton Dickinson	555663	Surface
158Gd	CD33	WM53	0.5	Becton Dickinson	555449	Surface
160Gd	CD14	M5E2	1	Becton Dickinson	555396	Surface
161Dy	CD274	29E.2A3	5	BioLegend	329702	Surface
162Dy	FoxP3	PCH101	4	eBioscience	14-4776-82	Intracellular
163Dy	CD159c (NKG2C)	REA205	4	Miltenyi Biotec	130-095-212	Surface
164Dy	CD161	DX12	4	Becton Dickinson	556079	Surface
165Ho	CD127	A019D5	2	BioLegend	351302	Surface
166Er	CD34	581	4	Becton Dickinson	555820	Surface
167Er	CD38	HIT2	4	BioLegend	303502	Surface
169Tm	CD25	M-A251	4	BioLegend	356102	Surface
170Er	CD3	UCHT1	1	BioLegend	300402	Surface
171Yb	Granzyme B	GB11	0.25	Acris	SM1806P	Intracellular
172Yb	CD197 (CCR7)	G043H7	2	BioLegend	353202	Surface
173Yb	TCR Vα7.2	3C10	4	BioLegend	351702	Surface
174Yb	HLA-DR	L243	4	Becton Dickinson	556642	Surface
175Lu	CD279 (PD-1)	EH12.2H7	8	BioLegend	329902	Surface
176Yb	CD56	NCAM16.2	1	Becton Dickinson	559043	Surface
209Bi	CD57	NK-1	0.5	Becton Dickinson	555618	Surface

<u>Notes</u>: Antibodies were purchased in a purified, carrier-free unlabelled format from the indicated suppliers and conjugated with the indicated metal isotope by the Ramaciotti Facility for Human Systems Biology (The University of Sydney, Australia) using the Maxpar Antibody Labelling Kit (Fluidigm). All antibodies were titrated before inclusion in the panel.

Supplementary Table 4. Cell subset percentages included in SAM analysis

Percentages
B cells (% live cells)
Total monocytes (% live cells)
CD16^{+} γδ-T cells (% γδ-T cells)
CD16^{+} mDC (% mDC)
CD16^{+} monocytes (% total monocytes)
CD16^{+} NK cells (% NK cells)
CD161$^{low/-}$ Vα7.2^{+} CD8^{+} T cells (% live cells)
Tim3^{+} CD8^{+} T cells (% CD8^{+} T cells)
CD27^{+} CD4^{+} T cells (% CD4^{+} T cells)
CD27^{+} CD8^{+} T cells (% CD8^{+} T cells)
CD27^{+} γδ-T cells (% γδ-T cells)
CD27^{+} γδ-T cells (% live cells)
CD27^{+} B cells (% B cells)
Classical monocytes (% live cells)
CD3^{-} cells (% live cells)
CD3^{+}CD33^{-} cells (% live cells)
CD34^{+}CD3^{-} cells (% live cells)
CD38^{+} CD4^{+} T cells (% CD4^{+} T cells)
CD38^{+} CD8^{+} MAIT cells (% CD8^{+} MAIT cells)
CD38^{+} HLA-DR^{+} EM CD4^{+} T cells (% CD4^{+} T cells)
CD38^{+} HLA-DR^{+} EM CD4^{+} T cells (% EM CD4^{+} T cells)
CD38^{+} HLA-DR^{+} EM CD8^{+} T cells (% CD8^{+} T cells)
CD38^{+} HLA-DR^{+} EM CD8^{+} T cells (% EM CD8^{+} T cells)
CD38^{+} non-classical monocytes (% non-classical monocytes)
CD38^{+}GzmB^{+} CM CD8^{+} T cells (% CD8^{+} T cells)
CD38^{+}GzmB^{+} CM CD8^{+} T cells (% CM CD8^{+} T cells)
CD38^{+} HLA-DR^{+} CD4^{+} T cells (% CD4^{+} T cells)
CD3^{-}CD56^{-}CD14^{+}CD38^{+}HLA-DR^{-} cells (% live cells)
CD4:CD8 ratio
CD4^{+} T cells (% live)
CD4^{+}CD8^{+} T cells (% CD3^{+} cells)
CD45RA^{+} γδ-T cells (% γδ-T cells)
CD45RA^{+}GzmB^{+}CD27^{-} γδ-T cells (% γδ-T cells)
CD57^{+} CD4^{+} T cells (% CD4^{+} T cells)
CD57^{+} CD8^{+} T cells (% CD8^{+} T cells)
CD57^{+} CD8^{+} T cells (% live cells)
CD57^{+}GzmB^{+} CD8^{+} T cells (% CD8^{+} T cells)
CD57^{+} NK cells (% NK cells)
CD57^{+}CD38^{+} CD8^{+} T cells (% CD8^{+} T cells)
CD57^{+}NKG2C^{+} NK cells (% NK cells)
CD69^{+} CD8^{+} MAIT cells (% CD8^{+} MAIT cells)
CD69^{+} NK cells (% NK cells)
CD8^{+} T cells (% live)

Table S4 continued over page

CD86$^+$ monocytes (% total monocytes)
CD86$^+$ classical monocytes (% classical monocytes)
CD86$^+$ intermediate monocytes (% intermediate monocytes)
CD86$^+$ non-classical monocytes (% non-classical monocytes)
CD86$^+$ mDCs (% mDCs)
CM CD4$^+$ T cells (% CD4$^+$ T cells)
CM CD4$^+$ T cells (% live cells)
CM CD8$^+$ T cells (% CD8$^+$ T cells)
EM CD4$^+$ T cells (% CD4$^+$ T cells)
EM CD8$^+$ T cells (% CD8$^+$ T cells)
γδ-T cells (% live cells)
GzmB$^+$ CD8$^+$ T cells (% CD8$^+$ T cells)
GzmB$^+$ CD4$^+$ T cells (% CD4$^+$ T cells)
GzmB$^+$ γδ-T cells (% γδ-T cells)
CD57$^+$GzmB$^+$ CD3$^+$CD56$^+$ cells (% CD3$^+$CD56$^+$ cells)
HLA-DR$^+$ NK cells (% NK cells)
HLA-DR$^+$CD38$^+$ CD8$^+$ MAIT cells (% CD8$^+$ MAIT cells)
HLA-DR$^+$CD38$^+$ CD8$^+$ T cells (% CD8$^+$ T cells)
ICOS$^+$ CD4$^+$ T cells (% CD4$^+$ T cells)
ICOS$^+$ HLADR$^+$CD38$^+$ CD4$^+$ T cells (% CD4$^+$ T cells)
Intermediate monocytes (% total monocytes)
CD8$^+$ MAIT cells (% Va7.2$^+$ CD8$^+$ T cells)
CD8$^+$ MAIT cells (% live cells)
Total MAIT cells (% live cells)
CD8$^+$ MAIT cells (% CD8$^+$ T cells)
mDC (% live cells)
Naive CD4$^+$ T cells (% CD4$^+$ T cells)
Naive CD8$^+$ T cells (% CD8$^+$ T cells)
NK cells (% live cells)
NKG2C$^+$ CD8$^+$ T cells (% CD8$^+$ T cells)
CD3$^+$CD56$^+$ cells (% CD3$^+$ cells)
CD3$^+$CD56$^+$ cells (% live cells)
Non-classical monocytes (% total monocytes)
PD-1$^+$ CD4$^+$ T cells (% CD4$^+$ T cells)
PD-1$^+$ CD8$^+$ T cells (% CD8$^+$ T cells)
PD-1$^+$ EM CD8$^+$ T cells (% CD8$^+$ T cells)
PD-1$^+$ EMRA CD8$^+$ T cells (% CD8$^+$ T cells)
pDC (% live cells)
PD-L1$^+$ classical monocytes (% classical monocytes)
CD45RA:CD45RO ratio on CD4$^+$ T cells (% CD4$^+$ T cells)
EMRA CD4$^+$ T cells (% CD4$^+$ T cells)
EMRA CD8$^+$ T cells (% CD8$^+$ T cells)
Tim3$^+$ intermediate monocytes (% intermediate monocytes)
Tregs (% live cells)
CD57$^+$PD-1$^+$CD38$^+$ CD4$^+$ T cells (% CD4$^+$ T cells)

Supplementary Table 5. Absolute cell counts included in SAM analysis

Absolute counts (x10^{9}/L)
B cells
CD16^{-} NK cells
CD16^{+} γδ-T cells
CD16^{+} mDC
CD16^{+} NK cells
CD16^{+} total monocytes
CD161$^{low/-}$ Vα7.2^{+} CD8^{+} T cells
CD27^{+} CD4^{+} T cells
CD27^{+} CD8^{+} T cells
CD27^{+} γδ-T cells
CD34^{+}CD3^{-} cells
CD38^{+} CD8^{+} MAIT cells
CD38^{+} non-classical monocytes
CD38^{+}GzmB^{+} CM CD8^{+} T cells
CD4^{+} T cells
CD45RA^{+} γδ-T cells
CD45RA^{+}GzmB^{+}CD27^{-} γδ-T cells
CD4^{+}CD8^{+} T cells
CD27^{+} B cells
Total MAIT cells
CD57^{+} CD4^{+} T cells
CD57^{+} CD8^{+} T cells
CD57^{+} NK cells
CD57^{+}CD38^{+} CD8^{+} T cells
CD38^{+} CD4^{+} T cells
CD57^{+}GzmB^{+} CD8^{+} T cells
CD57^{+}GzmB^{+} CD3^{+}CD56^{+} cells
CD57^{+}NKG2C^{+} NK cells
CD57^{+}PD-1^{+}CD38^{+} CD4^{+} T cells
CD69^{+} CD8^{+} MAIT cells
CD69^{+} NK cells
CD8^{+} T cells
CD8^{+} MAIT cells
CD86^{+} total monocytes
CD86^{+} classical monocytes
CD86^{+} intermediate monocytes
CD86^{+} mDC
CD86^{+} non-classical monocytes
Classical monocytes
CM CD4^{+} T cells
CM CD8^{+} T cells

Table S5 continued over page

EM $CD4^+$ T cells
EM $CD8^+$ T cells
γδ-T cells
$GzmB^+$ γδ-T cells
$GzmB^+$ $CD4^+$ T cells
$GzmB^+$ $CD8^+$ T cells
HLA-DR^+ NK cells
HLA-$DR^+$$CD38^+$ $CD4^+$ T cells
HLA-$DR^+$$CD38^+$ $CD8^+$ MAIT cells
HLA-$DR^+$$CD38^+$ $CD8^+$ T cells
HLA-$DR^+$$CD38^+$ EM $CD4^+$ T cells
HLA-$DR^+$$CD38^+$ EM $CD8^+$ T cells
$ICOS^+$ $CD4^+$ T cells
$ICOS^+$ $HLADR^+$$CD38^+$ $CD4^+$ T cells
Intermediate monocytes
Total lymphocytes*
mDC
Total monocytes*
Naïve $CD4^+$ T cells
Naïve $CD8^+$ T cells
NK cells
$NKG2C^+$ $CD8^+$ T cells
$CD3^+$$CD56^+$ cells
Non-classical monocytes
PD-1^+ $CD8^+$ T cells
PD-1^+ $CD4^+$ T cells
PD-1^+ EM $CD8^+$ T cells
PD-1^+ EMRA $CD8^+$ T cells
pDC
PD-$L1^+$ classical monocytes
EMRA $CD4^+$ T cells
EMRA $CD8^+$ T cells
$Tim3^+$ $CD8^+$ T cells
$Tim3^+$ intermediate monocytes
Tregs
WBC*

*derived directly from automated full blood analyser

Supplementary Table 6. Characteristics of $CD8^{high}$ and $CD8^{low}$ HSCT recipients with HCMV reactivation

Characteristic	$CD8^{low}$ (n=7)	$CD8^{high}$ (n=10)	p value
**LR : HR**	1 : 6	4 : 6	0.3382
**Age (median (range))**	47 (18 – 67)	57.5 (18 – 62)	0.7937
**Sex (M : F)**	2 : 5	4 : 6	>0.9999
HCMV serostatus<br>D-/R+<br>D+/R+	 2 (29%)<br>5 (71%)	 3 (30%)<br>7 (70%)	>0.9999
Underlying diagnosis<br>AML<br>ALL<br>MDS<br>SAA<br>MPD	 3<br>1<br>0<br>3<br>0	 4<br>4<br>1<br>0<br>1	ND
Conditioning<br>MAC<br>RIC	 1 (14%)<br>6 (86%)	 4 (40%)<br>6 (60%)	0.3382
Stem cell source<br>Peripheral blood<br>Bone marrow	 4 (57%)<br>3 (43%)	 10 (100%)<br>0	**0.0515**
Donor type<br>MUD<br>Haploidentical<br>HLA-identical related	 4<br>0<br>3	 7<br>1<br>2	ND
**T cell depletion**	7 (100%)	6 (60%)	0.1029
Acute GvHD<br>Overall (grade II-IV)<br>Severe (grade III-IV)	 2 (29%)<br>0	 2 (20%)<br>0	>0.9999
**EBV reactivation**	5 (71%)	5 (50%)	0.6221
**Relapse**	3 (43%)	3 (30%)	0.6437
**Death (1st year post-HSCT)**	2 (29%)	1 (10%)	0.5368
**Death (overall follow up)**	3 (43%)	3 (30%)	0.6437
**Peak HCMV copies/mL**	2690 (171-52740)	2108 (150-16100)	0.5999
**T3 sample (days post-HSCT)**	47 (39-62)	46.5 (43-60)	0.7917
**T3 sample (days post initial detection of HCMV reactivation)**	21 (7-28)	21 (7-42)	0.9434
**HCMV log_{10} AUC T3**	4.09 (3.20-5.35)	4.09 (3.20-4.96)	0.7611
**HCMV log_{10} AUC_{0-100}**	4.72 (3.69-5.54)	4.46 (3.32-5.28)	0.3773
**Antiviral treatment for HCMV reactivation (n patients)**	6 (86%)	7 (70%)	0.6029

Notes: HSCT recipients with HCMV reactivation were stratified into '$CD8^{high}$' and '$CD8^{low}$' groups by the presence or absence of an elevated $CD8^{+}$ T-cell dominated immune signature at the peak of reactivation (T3). Data show absolute number (n, %) or median with range. Death (overall follow up) refers to overall follow-up period of 728 (78-1242) days post-

transplant (for $CD8^{high}$ and $CD8^{low}$ patients). Note that HCMV reactivators without a T3 sample (n=2) are not included. p values represent statistical comparisons between '$CD8^{high}$' and '$CD8^{low}$' groups; $p<0.05$ considered significant. Two-sided Fisher's exact test used for categorical variables; two-tailed Mann-Whitney test used for continuous variables. ALL (acute lymphoblastic leukaemia), AML (acute myeloid leukaemia), AUC (area under the curve), AUC_{0-100} (HCMV DNA area under the curve in the first 100 days post-HSCT), D (donor), EBV (Epstein-Barr Virus), GvHD (graft-versus-host disease), HCMV (human cytomegalovirus), HR (high-level HCMV reactivation; 830-53,000 peak HCMV DNA copies/mL), LR (low-level HCMV reactivation; <250 peak HCMV DNA copies/mL), MAC (myeloablative conditioning), MDS (myelodysplastic syndrome), MPD (myeloproliferative disorder), MUD (matched unrelated donor), ND (not determined), R (recipient), RIC (reduced intensity conditioning), SAA (severe aplastic anaemia), T3 (peak of HCMV DNAemia timepoint).
