# Supplementary material for: Immunoprofiling reveals cell subsets associated with the trajectory of cytomegalovirus reactivation post stem cell transplantation
Source: Nat Commun. 2022 May 11;13:2603. doi: 10.1038/s41467-022-29943-9 (PMC9095831; doi:10.1038/s41467-022-29943-9)
Supplement: Supplementary file 2 — Reporting Summary [file 41467_2022_29943_MOESM2_ESM.pdf]

nature portfolio

Corresponding author(s): Barry Slobedman

Last updated by author(s): Feb 24, 2022

Reporting Summary

Nature Portfolio wishes to improve the reproducibility of the work that we publish. This form provides structure for consistency and transparency in reporting. For further information on Nature Portfolio policies, see our Editorial Policies and the Editorial Policy Checklist.

Statistics

For all statistical analyses, confirm that the following items are present in the figure legend, table legend, main text, or Methods section.

n/a	Confirmed	
☐	☒	The exact sample size (*n*) for each experimental group/condition, given as a discrete number and unit of measurement
☐	☒	A statement on whether measurements were taken from distinct samples or whether the same sample was measured repeatedly
☐	☒	The statistical test(s) used AND whether they are one- or two-sided<br>*Only common tests should be described solely by name; describe more complex techniques in the Methods section.*
☒	☐	A description of all covariates tested
☐	☒	A description of any assumptions or corrections, such as tests of normality and adjustment for multiple comparisons
☐	☒	A full description of the statistical parameters including central tendency (e.g. means) or other basic estimates (e.g. regression coefficient) AND variation (e.g. standard deviation) or associated estimates of uncertainty (e.g. confidence intervals)
☐	☒	For null hypothesis testing, the test statistic (e.g. *F*, *t*, *r*) with confidence intervals, effect sizes, degrees of freedom and *P* value noted<br>*Give P values as exact values whenever suitable.*
☒	☐	For Bayesian analysis, information on the choice of priors and Markov chain Monte Carlo settings
☒	☐	For hierarchical and complex designs, identification of the appropriate level for tests and full reporting of outcomes
☒	☐	Estimates of effect sizes (e.g. Cohen's *d*, Pearson's *r*), indicating how they were calculated

Our web collection on statistics for biologists contains articles on many of the points above.

Software and code

Policy information about availability of computer code

Data collection	Mass cytometry acquisition: CyTOF 2 Helios upgraded mass cytometer (Fluidigm), CyTOF Software version 6.7.1014. Sysmex 1800 full blood analyser used to measure total white blood cell, lymphocyte and monocyte counts x10^9/L peripheral blood.
Data analysis	FlowJo version 10.0.7 (Tree Star, Inc.) for manual gating of .fcs files. GraphPad Prism Software version 8.2.1 for graphs and statistics. Multi Experiment Viewer 4.9.0 (TM4) for significance analysis of microarrays. CyTOF Software version 6.7.1014 for normalisation of FCS files.

For manuscripts utilizing custom algorithms or software that are central to the research but not yet described in published literature, software must be made available to editors and reviewers. We strongly encourage code deposition in a community repository (e.g. GitHub). See the Nature Portfolio guidelines for submitting code & software for further information.

Data

Policy information about availability of data

All manuscripts must include a data availability statement. This statement should provide the following information, where applicable:

- Accession codes, unique identifiers, or web links for publicly available datasets
- A description of any restrictions on data availability
- For clinical datasets or third party data, please ensure that the statement adheres to our policy

The data underlying all tables and figures are provided in the Source Data file. The data generated during and/or analysed during the current study are available from the corresponding author (B.S.) on reasonable request. Source data are provided with this paper.

Field-specific reporting

Please select the one below that is the best fit for your research. If you are not sure, read the appropriate sections before making your selection.

- [x] Life sciences
- [ ] Behavioural & social sciences
- [ ] Ecological, evolutionary & environmental sciences

For a reference copy of the document with all sections, see nature.com/documents/nr-reporting-summary-flat.pdf

Life sciences study design

All studies must disclose on these points even when the disclosure is negative.

Sample size	The number of patients studied (n=35) reflects the number of individuals undergoing HSCT who consented to participate in the study and had PBMC samples available for analysis. Sample size was not pre-determined. Patients were recruited sequentially from Westmead Hospital between 2015-2017. The number of patients in each subgroup (SN n=11, SP-NR n=5, LR n=6, HR n=13) emerged on the basis of pre-transplant HCMV serostatus and magnitude of post-transplant HCMV DNAemia in the recruited population.
Data exclusions	No data were excluded. For analyses involving the 'CD8 high' and 'CD8 low' groups, two patients (n=1 HR, n=1 LR) who experienced HCMV reactivation but lacked a T3 PBMC sample could not be categorised into 'CD8 high' or 'CD8 low' groups, as these groups were defined based on the immune profile seen at T3. Consequently these patients were not included in the subset of analyses involving the CD8 high/low groups
Replication	Mass cytometry on patient samples was not repeated due to the limited volume of clinical material. Biological replicates ranged from 5 to 13 independent samples per group per timepoint (see Suppl Table 2), depending on the number of samples available. Samples were stained across 8 batches containing a mix of patient groups and a healthy donor control. The study did not involve in vitro functional assays.
Randomization	Randomisation was not relevant to our study design as our goal was to compare immune reconstitution across four different patient groups, defined as follows: The patients were retrospectively divided into four groups according to pre-transplant HCMV serostatus and magnitude of post-transplant HCMV reactivation. The 'Seronegative' (SN; n=11) group were HCMV-seronegative recipients with seronegative donors (D-/R-), and had no detected HCMV DNAemia. The 'Seropositive No Reactivation' (SP-NR; n=5) group were HCMV-seropositive recipient (R+) and/or donor (D+) patients with no documented HCMV reactivation in the first 100 days post-HSCT. 'Low-level reactivators' (LR; n=6) developed HCMV reactivation to <250 peak copies/mL. 'High-level reactivators' (HR; n=13) developed HCMV reactivation to >830 peak copies/mL. No patients in the study had peak HCMV titres between 250-830 copies/mL.
Blinding	Blinding was not relevant to our study as we retrospectively assigned patients into four different groups, based upon their HCMV serostatus and any subsequent post-transplant HCMV reactivation. However, at the time of collection and initial processing of patient samples, we were blinded to these parameters.

Reporting for specific materials, systems and methods

We require information from authors about some types of materials, experimental systems and methods used in many studies. Here, indicate whether each material, system or method listed is relevant to your study. If you are not sure if a list item applies to your research, read the appropriate section before selecting a response.

Materials & experimental systems

n/a	Involved in the study	
	x	Antibodies
x		Eukaryotic cell lines
x		Palaeontology and archaeology
x		Animals and other organisms
	x	Human research participants
x		Clinical data
x		Dual use research of concern

Methods

n/a	Involved in the study	
x		ChIP-seq
	x	Flow cytometry
x		MRI-based neuroimaging

Antibodies

Antibodies used	Antibodies were purchased from commercial suppliers in a purified, unlabelled format and were conjugated with the indicated metal isotope by the Ramaciotti Facility for Human Systems Biology using the Maxpar Antibody Labelling Kit (Fluidigm). The following antibody panel was used for mass cytometry:<br><br>89Y-CDK1: clone Cdc2 p34 (17), 8 µg/mL, Santa Cruz Biotechnology, catalogue number: sc-54<br>115In-CD11c: clone Bu15, 1 µg/mL, BioLegend, catalogue number: 337202<br>141Pr-CD27: clone M-T271, 1 µg/mL, Becton Dickinson, catalogue number: 555439<br>142Nd-CD19: clone HIB19, 1 µg/mL, Becton Dickinson, catalogue number: 555410<br>143Nd-CD45RA: clone HI100, 2 µg/mL, Becton Dickinson, catalogue number: 555486

144Nd-CD69: clone FN50, 3 µg/mL, BioLegend, catalogue number: 310939
145Nd-CD4: clone RPA-T4, 2 µg/mL, Becton Dickinson, catalogue number: 555344
146Nd-CD8A: clone RPA-T8, 8 µg/mL, BioLegend, catalogue number: 301002
147Sm-CD20: clone 2H7, 4 µg/mL, BioLegend, catalogue number: 302302
148Nd-CD16: clone 3G8, 1 µg/mL, Becton Dickinson, catalogue number: 556617
149Sm-TIM3: clone 7D3, 1 µg/mL, Becton Dickinson, catalogue number: 565768
150Nd-TCR γ/δ: clone B1, 8 µg/mL, Becton Dickinson, catalogue number: 555715
151Eu-CD278: clone DX29, 4 µg/mL, Becton Dickinson, catalogue number: 557801
152Sm-CD45RO: clone UCHL1, 8 µg/mL, Becton Dickinson, catalogue number: 555491
153Eu-CD304: clone 12C2, 1 µg/mL, BioLegend, catalogue number: 354502
154Sm-CD163: clone GHI/61, 6 µg/mL, Becton Dickinson, catalogue number: 556017
155Gd-CD314 (NKG2D): clone 1D11, 6 µg/mL, Becton Dickinson, catalogue number: 552866
156Gd-CD86: clone IT2.2, 2 µg/mL, Becton Dickinson, catalogue number: 555663
158Gd-CD33: clone WM53, 0.5 µg/mL, Becton Dickinson, catalogue number: 555449
160Gd-CD14: clone M5E2, 1 µg/mL, Becton Dickinson, catalogue number: 555396
161Dy-CD274: clone 29E.2A3, 5 µg/mL, BioLegend, catalogue number: 329702
162Dy-FoxP3: clone PCH101, 4 µg/mL, eBioscience, catalogue number: 14-4776-82
163Dy-CD159c (NKG2C): clone REA205, 4 µg/mL, Miltenyi Biotec, catalogue number: 130-095-212
164Dy-CD161: clone DX12, 4 µg/mL, Becton Dickinson, catalogue number: 556079
165Ho-CD127: clone A019D5, 2 µg/mL, BioLegend, catalogue number: 351302
166Er-CD34: clone 581, 4 µg/mL, Becton Dickinson, catalogue number: 555820
167Er-CD38: clone HIT2, 4 µg/mL, BioLegend, catalogue number: 303502
169Tm-CD25: clone M-A251, 4 µg/mL, BioLegend, catalogue number: 356102
170Er-CD3: clone UCHT1, 1 µg/mL, BioLegend, catalogue number: 300402
171Yb-Granzyme B: clone GB11, 0.25 µg/mL, Acris, catalogue number: SM1806P
172Yb-CD197 (CCR7): clone G043H7, 2 µg/mL, BioLegend, catalogue number: 353202
173Yb-TCR Vα7.2: clone 3C10, 4 µg/mL, BioLegend, catalogue number: 351702
174Yb-HLA-DR: clone L243, 4 µg/mL, Becton Dickinson, catalogue number: 556642
175Lu-CD279 (PD-1): clone EH12.2H7, 8 µg/mL, BioLegend, catalogue number: 329902
176Yb-CD56: clone NCAM16.2, 1 µg/mL, Becton Dickinson, catalogue number: 559043
209Bi-CD57: clone NK-1, 0.5 µg/mL, Becton Dickinson, catalogue number: 555618

Validation

Antibodies were conjugated with metal isotopes by the Ramaciotti Facility for Human Systems Biology. Titration of conjugated antibodies for mass cytometry was performed on cryopreserved healthy donor PBMCs using appropriate controls. All antibodies were validated, pre-titered and supplied in per-test amounts by the Ramaciotti Facility for Human Systems Biology Mass Cytometry Reagent Bank (The University of Sydney).

The following are general statements regarding antibody validation from the manufacturers' websites:

BD (https://www.bdbiosciences.com/en-us/products/reagents/flow-cytometry-reagents/research-reagents/quality-and-reproducibility): "The specificity is confirmed using multiple methodologies that may include a combination of flow cytometry, immunofluorescence, immunohistochemistry or western blot to test staining on a combination of primary cells, cell lines or transfectant models. All flow cytometry reagents are titrated on the relevant positive or negative cells.","SOPs and guidelines according to ISO requirements are strictly followed to ensure that subsequent batches of the reagent provide reproducible results to help you obtain experimental success and confidence in your research. This includes testing side by side with prior batches as reference so that the new batch provides consistent performance in the intended application."

Biolegend (https://www.biolegend.com/en-us/bio-bits/highly-specific-validated-antibodies): "Antibody validation is a critical step in the journey towards obtaining consistent reproducibility in science. To ensure they are both specific and sensitive, we validate our antibodies through a variety of methods including: Testing on multiple cell and tissue types with a variety of known expression levels. Validation in multiple applications as a cross-check for specificity and to provide additional clarity for researchers. Comparison to existing antibody clones. Using cell treatments to modulate target expression, such as phosphatase treatment to ensure phospho-antibody specificity." (https://www.biolegend.com/en-us/quality/quality-control): "Flow Cytometry Reagents: Specificity testing of 1-3 target cell types with either single- or multi-color analysis (including positive and negative cell types). Once specificity is confirmed, each new lot must perform with similar intensity to the in-date reference lot. Brightness (MFI) is evaluated from both positive and negative populations. Each lot product is validated by QC testing with a series of titration dilutions."

Miltenyi biotec (https://www.miltenyibiotec.com/AU-en/products/macs-antibodies/antibody-validation.html):
"REAfinity Recombinant Antibodies are based on three pillars of validation: reproducibility, specificity, and sensitivity. Please find more detailed information for each validation method below:
1. Antibody reproducibility and consistency
"Recombinant antibodies: The nature of our REAfinity Recombinant Antibodies ensures reproducibility since they don't have any Immunoglobulin impurities and don't show background signal due to a mutated Fc region.
Highly purified antibody products: Recombinant antibodies ensure high lot-to-lot consistency as compared to traditional hybridoma technology. Mass spectrometry analysis of the purified recombinant antibodies confirms the improved purity of antibody products.
Purification of final product: After conjugation of antibodies, any unbound fluorochromes and antibodies are removed to purify the final product.
Lot-to-lot consistent performance: In addition, all antibodies are tested for lot-to-lot consistency at two stages, during the antibody raw material production as well as the fluorochrome-conjugation process. This includes purification steps to remove unconjugated fluorochromes and antibodies from the mixture, as well as side-by-side comparisons with previous batches.
2. Validation of antibody specificity

During development of an antibody, a suitable test to verify specificity of the clone is performed. Several approaches are possible. Below you will find a list of methods we typically perform.
Counterstaining. Knockout of target protein. Epitope competition assay. siRNA knockdown. Stimulation of cells. Overexpression of target protein. Binding to purified antigen (latex bead coating). Cross-reactivity.
3. Antibody sensitivity
"How to validate an antibody after purchasing? Validation is of course not solely the responsibility of the supplier because antibody validation methods and antibody validation protocols are highly dependent on the intended application and experimental setup. For this reason, we recommend thoroughly researching the most suited validation methods for your application."

CDK1 (Santa Cruz): https://www.scbt.com/p/cdc2-p34-antibody-17
Granzyme B (Acris/Origene): https://www.origene.com/catalog/antibodies/primary-antibodies/sm1806p/granzyme-b-gzmb-mouse-monoclonal-antibody-clone-id-gb11
FoxP3 (thermofisher/ebioscience) https://www.thermofisher.com/antibody/product/FOXP3-Antibody-clone-PCH101-Monoclonal/14-4776-82: "This PCH101 antibody has been tested by immunoblotting (WB) (1-5 µg/mL) of normal human peripheral blood leukocytes. This PCH101 antibody has been tested by immunohistochemistry of formalin-fixed paraffin embedded tissue using low pH antigen retrieval and can be used at less than or equal to 10 µg/mL. It is recommended that the antibody be carefully titrated for optimal performance in the assay of interest. This Antibody was verified by Relative expression to ensure that the antibody binds to the antigen stated.

Human research participants

Policy information about studies involving human research participants

Population characteristics

Adult allogeneic haematopoietic stem cell transplant recipients (n=35), age 18-70 years, male:female 20:15, indications for transplant were haematological malignancies or severe aplastic anaemia. Stem cell source was mobilised peripheral blood (n=32) or bone marrow (n=3). 14 received myeloablative conditioning, 21 received reduced intensity conditioning. 20/35 received T cell depletion. 22/35 had matched unrelated donors, 4/35 haploidentical, 9/35 HLA-identical related donors. 19/35 had HCMV DNAemia detected in first 100 days post-HSCT. Table 1 provides a further outline of patient characteristics.

Recruitment

Participants were patients presenting for treatment (haematopoietic stem cell transplant procedures) at Westmead Hospital (Sydney, Australia) and were recruited sequentially between 2015-2017 with blood samples collected weekly in the first 100 days post-transplant. In the present mass cytometry study, a criteria for inclusion was the availability of cryopreserved PBMC samples corresponding with at least two time-points in our retrospective study design (T1,T2,T3,T4), thus there may be a potential bias towards patients who survived to provide sufficient samples.

Ethics oversight

The study was approved by the University of Sydney and Western Sydney Local Health District ethics committees (Project Number 2014/440)

Note that full information on the approval of the study protocol must also be provided in the manuscript.

Flow Cytometry

Plots

Confirm that:

- [x] The axis labels state the marker and fluorochrome used (e.g. CD4-FITC).
- [x] The axis scales are clearly visible. Include numbers along axes only for bottom left plot of group (a 'group' is an analysis of identical markers).
- [x] All plots are contour plots with outliers or pseudocolor plots.
- [ ] A numerical value for number of cells or percentage (with statistics) is provided.

Methodology

Sample preparation

Peripheral blood in EDTA vacutainers was collected from adult HSCT recipients in the first 100 days post-transplant. Peripheral blood mononuclear cells (PBMC) were isolated by Ficoll-Paque PLUS (GE Healthcare) density-gradient centrifugation and cryopreserved in freezing media (70% v/v RPMI-1640, 20% v/v FBS, 10% v/v DMSO). For mass cytometry staining, PBMCs were resuscitated by thawing in a 37 degrees Celsius waterbath and washed with warm RPMI-1640 supplemented with 10% (v/v) FBS and 1:10000 (v/v) Pierce Universal Cell Nuclease (Thermo Fisher Scientific). Cells were then washed with warm RPMI-1640 containing 10% FBS, then with serum-free RPMI-1640. Cells were then incubated with 1.25 µM Cell-ID cisplatin (Fluidigm) in serum-free RPMI-1640 for 3 min at RT, followed by immediate quenching with RPMI-1640 containing 10% FBS. Cells were washed once with FACS buffer (DPBS with 1% FBS and 0.01 M EDTA). For surface staining, cells were incubated in 50 µL surface antibody cocktail (in FACS buffer) for 30 min at 4 degrees C. Cells were washed twice with FACS buffer, then fixed and permeabilised by incubation in 1X FoxP3 Fixation/Permeabilisation buffer (eBioscience) for 45 min at 4 degrees C. Cells were then washed twice with 1X permeabilisation buffer (eBioscience) and stained with 50 µL intracellular antibody cocktail (in 1X permeabilisation buffer) for 45 min at 4 degrees C. After washing with 1X permeabilisation buffer and FACS buffer, cells were fixed in 4% PFA (prepared in PBS) containing 0.125 µM Cell-ID Iridium-191/193 nucleic acid intercalator for 20 min RT, then stored at 4 degrees C overnight or for up to one week prior to acquisition. Prior to CyTOF acquisition, cells were washed once in FACS buffer, once in DPBS and twice in MilliQ water. The pellet was resuspended at 0.8 x10^6 cells/mL in 1:10 (v/v) EQ Four Element Calibration Beads (Fluidigm) in MilliQ water, and filtered through a 0.35 µm nylon cell strainer snap-cap immediately before acquisition.

Instrument	CyTOF 2 Helios upgraded mass cytometer (Fluidigm)
Software	CyTOF Software version 6.7.1014 for normalisation of FCS files. FlowJo version 10.0.7 (Tree Star, Inc.) for manual gating of immune subsets.
Cell population abundance	This study did not involve cell sorting. Abundance of various cell subsets as a percentage of PBMCs as measured by mass cytometry and manual gating is reported throughout the manuscript.
Gating strategy	Single events were distinguished from doublets and debris on the basis of DNA intercalator staining and event length. Cisplatin-negative cells (live cells) were selected, then EQ beads (Eu151+ Ce140+) excluded. Major immune subsets were then identified using the gating strategy in Supplementary Figure 2. A list of all subsets analysed is shown in Supplementary Table 4 and Supplementary Table 5. Analysis of cell subset percentages included both percent of live cells (% live) and/or percent of parent subset. Percentages of live cells (% live) were derived from the "live cells" gate.

☒ Tick this box to confirm that a figure exemplifying the gating strategy is provided in the Supplementary Information.
